# Genome-Wide Gene-Set Analysis Identifies Molecular Mechanisms Associated with ALS

**DOI:** 10.3390/ijms24044021

**Published:** 2023-02-16

**Authors:** Christina Vasilopoulou, Sarah L. McDaid-McCloskey, Gavin McCluskey, Stephanie Duguez, Andrew P. Morris, William Duddy

**Affiliations:** 1 Personalised Medicine Centre, School of Medicine, Ulster University, Londonderry BT47 6SB, UK; 2 Centre for Genetics and Genomics Versus Arthritis, Centre for Musculoskeletal Research, University of Manchester, Manchester M13 9PT, UK

**Keywords:** amyotrophic lateral sclerosis (ALS), ALS pathology, gene-set analysis, functional genomics, GWAS

## Abstract

Amyotrophic lateral sclerosis (ALS) is a fatal late-onset motor neuron disease characterized by the loss of the upper and lower motor neurons. Our understanding of the molecular basis of ALS pathology remains elusive, complicating the development of efficient treatment. Gene-set analyses of genome-wide data have offered insight into the biological processes and pathways of complex diseases and can suggest new hypotheses regarding causal mechanisms. Our aim in this study was to identify and explore biological pathways and other gene sets having genomic association to ALS. Two cohorts of genomic data from the dbGaP repository were combined: (a) the largest available ALS individual-level genotype dataset (N = 12,319), and (b) a similarly sized control cohort (N = 13,210). Following comprehensive quality control pipelines, imputation and meta-analysis, we assembled a large European descent ALS-control cohort of 9244 ALS cases and 12,795 healthy controls represented by genetic variants of 19,242 genes. Multi-marker analysis of genomic annotation (MAGMA) gene-set analysis was applied to an extensive collection of 31,454 gene sets from the molecular signatures database (MSigDB). Statistically significant associations were observed for gene sets related to immune response, apoptosis, lipid metabolism, neuron differentiation, muscle cell function, synaptic plasticity and development. We also report novel interactions between gene sets, suggestive of mechanistic overlaps. A manual meta-categorization and enrichment mapping approach is used to explore the overlap of gene membership between significant gene sets, revealing a number of shared mechanisms.

## 1. Introduction

Amyotrophic lateral sclerosis (ALS) is the most common type of motor neuron disorder, characterized by the loss of both upper and lower motor neurons. This progressive motor neuron disease causes swallowing problems, paralysis, and eventually, death from neuromuscular respiratory failure [1,2,3]. Patients normally live for two to five years following the onset of symptoms, with 5–10% living for more than ten years [1,2,4]. ALS can affect individuals of any age; however, its peak onset is at 54–67 years of age [2,3,5,6]. Our current understanding of the etiology, genetic architecture and the underlying biological mechanisms of ALS is still elusive, slowing the development of prevention and treatment.

In recent years, genome-wide association studies (GWAS) have enabled the discovery of numerous associations of single nucleotide polymorphisms (SNPs) to complex diseases, including ALS. Previous genetic inheritance and genome-wide studies have identified numerous variants mapped to 46 genes as monogenic causes of ALS [7,8,9,10,11,12]. In European ancestry populations, the most common monogenic cause is the intronic hexanucleotide GGGGCC (G4C2) repeat expansion (HRE) in the *C9ORF72* gene [13,14]. Cu/Zn superoxide dismutase 1 *SOD1*, fused in sarcoma *FUS*, and transactive response DNA-binding protein of 43 kD *TARDBP/TDP-43* are further genes associated with ALS with great reproducibility [7]. However, genotype–phenotype studies have not fully elucidated the genetic contribution to familial and sporadic ALS. While a monogenic cause can be identified in around two-thirds of familial ALS cases [7,8], the majority of sporadic cases have no genetic cause identified [14,15]. Furthermore, it has been proposed that standard GWAS approaches are unlikely to fully unravel the genetic architecture of ALS and present a number of challenges and limitations [16]. One of the reasons for this is that GWAS is a single-marker analysis in which the contribution of each SNP is tested independently. Under this hypothesis, association p-values must be adjusted using strict multiple-testing methods, such as Bonferroni, in order to control for family-wise type I errors (false positives). As a result, GWAS has limited power to detect potential risk variants with weak genetic effects, which fail to pass the multiple testing correction, leading to family-wise type II errors (false negatives).

In complex diseases, such as ALS, multiple genes and biological pathways are expected to be implicated, making it necessary to understand the functional underpinnings of the disease. However, the gene products of the 30 or more known ALS-associated genes may interact with one another, participating in different molecular pathways. As such, traditional single-gene and single-marker studies are expected to present a number of challenges and limitations to the functional curation and interpretation [7,16,17]. Furthermore, univariate approaches, such as GWAS and single-gene analysis, do not consider the joint effects of multiple loci, events that are likely to be present in complex diseases such as ALS [16,18].

Understanding the functional mechanisms that underpin ALS has proven to be a challenging and complex task, made more difficult by the involvement of genes with diverse functional roles. Gene-set analysis (GSA) has been employed successfully by numerous genome-wide association studies as a method to understand the functional involvement of groups of genes in the phenotype of a complex disease. In gene-set analysis, individual SNPs are summarized into whole genes, taking into account multiple genetic associations, and genes are then summarized into gene sets [19]. A gene set is any group of genes that share a common attribute. A gene set can represent, inter alia, a biological pathway, a network module, or a group of interacting components. Each gene set is tested as a whole to investigate whether the gene set property is associated with the phenotype [19]. In a recent review [20], we describe and compare the results and the methodology of collected published gene-set analysis studies utilizing ALS GWAS cohorts which aim to identify functional pathways that are associated with ALS. We note several limitations of the collected ALS GSA studies, including the use of small ALS cohort sizes, and limited or under-documented genomic quality controls and GSA methodology. We further note the use of dimensionality reduction approaches applying arbitrary thresholds to SNPs or genes in order to ease functional interpretation, reducing reproducibility across different studies—as different thresholds may lead to different biological results and interpretations [21]—as well as risking the exclusion of false negative genes and gene sets or subtle associations.

**Figure 1:**
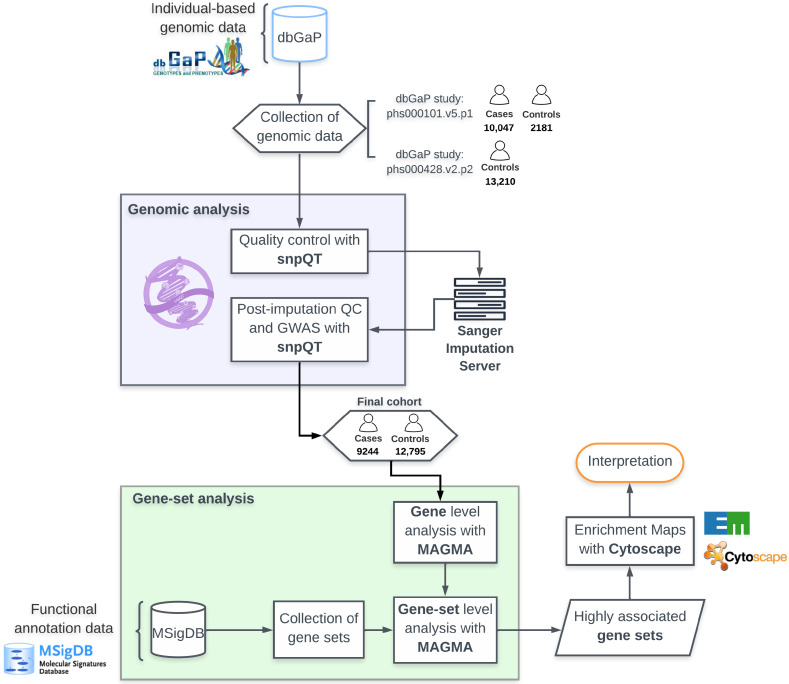
Overview of the ALS genome-wide gene-set analysis pipeline. Our analysis consisted of the following stages: (1) individual-level genomic data collection from the database of genotypes and phenotypes (dbGaP); (2) genomic analyses including QC using snpQT, batch effect correction strategies, preparation of the datasets for imputation using snpQT, imputation using the Sanger imputation server, post-imputation QC and GWAS using snpQT; (3) gene-set collection using molecular signatures database (MSigDB); (4) competitive and interaction gene-set analysis using the Multi-marker Analysis of GenoMic Annotation (MAGMA) software and (5) visualization and interpretation of the final results using enrichment maps in Cytoscape. QC: Quality control, GWAS: genome-wide association study.

The present study combines (a) the largest currently available ALS individual-level genomic study with a European descent from the dbGaP repository (N = 12,319) [9] and (b) an aging control cohort (N = 13,210), also from dbGaP, followed by comprehensive and careful quality control and batch effect correction strategies, which in our knowledge have not been previously utilized in an ALS genome-wide gene-set analysis study. Although there is no gold standard in genome-wide gene-set analysis, previous publications have demonstrated the increased power of competitive and mean-based gene-set analysis models [19,22]. By application of the MAGMA multi-model, which uses an aggregate test statistic combining multiple gene representations, the present analysis does not make an assumption about the underlying genetic architecture [22]. Two methods are employed, a combination that to our knowledge has not been used in previous ALS GSA studies: competitive and interaction gene-set analysis. Competitive gene-set analysis tests whether the genes within a given gene set are no more strongly associated with the phenotype than the genes that do not belong to this gene set [19]. We further investigate a more complex hypothesis that combinations of multiple gene sets are more highly associated with ALS than the individual gene sets, applying post hoc interaction gene-set analysis to all the significant gene sets [23]. Furthermore, we tested our gene-set analysis approach using the majority of the available gene sets from the molecular signatures database (MSigDB) [24,25]. Lastly, we sort associated gene sets into major biological categories after manual curation, and visualize the relationships between them using enrichment maps, where each node represents an associated gene set to ALS, and each edge represents the proportion of shared genes between two gene sets. The experimental design of the present study is shown in Figure 1.

The aim of this study is to identify statistically significant ALS-associated biological pathways using gene-set analysis on the largest available release of individual-level genomic ALS-control cohort data [9,26]. A large ALS-control cohort is created following detailed quality control and batch effect correction strategies [27,28]. Results are further visualized and interpreted using enrichment maps, and the relationships of the most significant gene sets are further investigated through interaction analysis. These analyses enable an in-depth exploration of the pathways associated with ALS.

## 2. Results

### 2.1. Gene-Level Meta-Analysis

Gene-level meta-analysis using MAGMA resulted in a total of 19,242 genes, from 22,039 samples of 9244 ALS cases and 12,795 healthy controls. The MAGMA multi-model gene-level results yielded 6 genes that reached high statistical significance based on false-discovery rate (FDR < 0.01) and 4 genes that passed the stricter multiple testing Bonferroni correction (alpha = 0.05; *p*-value $<2.58\times 10^{-6}$), shown in Table 1. The top 6 genes of our analysis include *MOB3B* (MOB kinase activator 3B; FDR = 1.77 $\times \;10^{-12}$), *IFNK* (interferon kappa; FDR = 3.98 $\times \;10^{-10}$), *C9ORF72* (chromosome 9 open reading frame 72; FDR = 2.50 $\times \;10^{-8}$), *UNC13A* (unc-13 homolog A; FDR = 1.14 $\times \;10^{-7}$), *ADARB1* (adenosine deaminase RNA specific B1; FDR = 0.001) and *KIF5A* (kinesin family member 5A; FDR = 0.008). The top three ALS-associated genes *MOB3B*, *IFNK* and *C9ORF72* have been reported in previous GWAS [29]. In addition, three of the six most strongly associated genes in this analysis have been established in previous studies as ALS-associated, including *C9ORF72* [30], *UNC13A* [31] and *KIF5A* [9]. These three genes were also reported as significantly associated with ALS in the original GWAS study reporting on these same data [9]. Our analysis also revealed two previously identified ALS-associated genes *TBK1* (TANK binding kinase) (FDR = 0.063) and *FUS* (fused in sarcoma; FDR = 0.155) [31] with a marginal statistical significance.

### 2.2. Gene-Set Analysis

We then carried out a gene-set analysis testing for the association of ALS to each of the 31,454 collected gene sets from the molecular signatures database (version 7.5). Twenty-four gene sets were associated to ALS with *p*-value < 0.05 and false discovery rate < 0.05 (Table 2).

These included gene sets belonging to various functional categories: the nervous system (the BioCarta CREB, DREAM, CK1, AGPCR and Shh pathways); the immune system (the BioCarta CSK, CTFR, TCR and VIP pathways); developmental pathways (the BioCarta Shh and mPR pathways); the cytoskeleton and cell cycle (the BioCarta Stathmin pathway); cell signaling (the GO cyclic nucleotide-dependent protein kinase activity, cyclic nucleotide binding, and the BioCarta IGF1R and AGPCR pathways); apoptosis and cell survival (the BioCarta BAD and IGF1R pathways); lipid metabolism and homeostasis (the BioCarta PPAR-alpha and CFTR pathways); and muscle tissue (the BioCarta IGF1R pathway). Gene-level summary statistics for the genes of each significant gene set are provided (Table S1).

**Figure 2:**
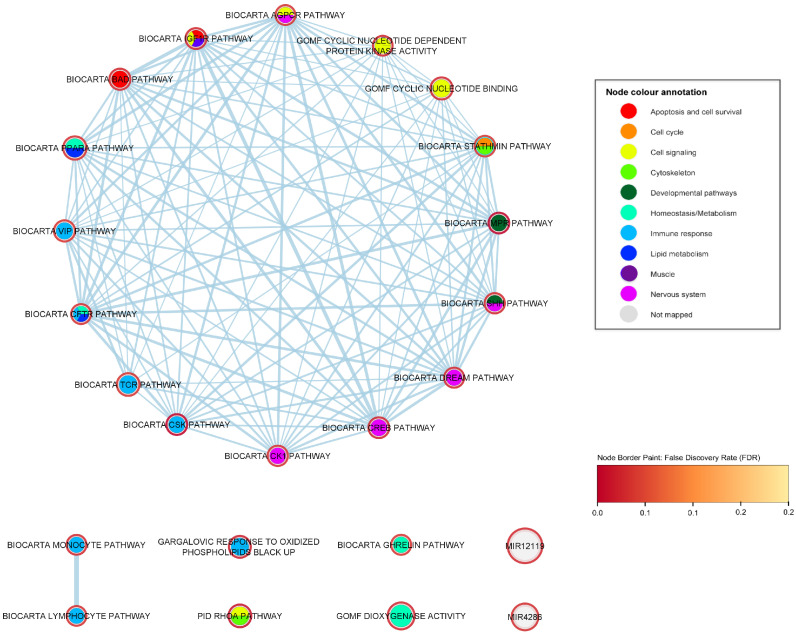
The ALS enrichment network that includes the 24 top ALS-associated gene sets (FDR < 0.05, *p*-value < 0.0029). We observe a highly interconnected cluster of 16 popular gene sets, that all contain a high degree within the generalized ALS network. The nodes within the cluster are arranged based on their main biological category, which we have classified as directly relevant to each node from related literature (right legend). The size of each node indicates the number of genes within each gene set. The statistical significance of each gene set/node is represented by the red color spectrum line outside each node (right legend). The edge that connects two nodes represents the gene overlap between them, and the edge width reflects the amount of this particular overlap.

Figure 2 shows an enrichment map containing only the strongly ALS-associated gene sets. Sixteen of these gene sets present a highly dense cluster, meaning that they often share genes with each other: their 16 nodes are connected to neighbors through 113 edges (representing shared gene membership). The eight nodes of the remaining gene sets have just one or no first neighbor among the strongly associated gene sets. The nervous system is the most represented functional category within the cluster, for which five pathways are associated with the disease.

To explore in greater depth the functional relationships of ALS-associated gene sets, we carried out an exhaustive enrichment map exploration based on *p*-value cut-off of 0.05 and FDR cut-off of 0.25. Enrichment maps are a way of representing the overlapping relationships between gene sets. This approach allows visualizing not only the direct overlap between the strongly ALS-associated gene sets described above (i.e., the gene sets which we would consider to be the primary observation of the study), but also their wider functional context, drawing in a total of 145 gene sets. The following sections first consider an enrichment map containing only the strongly ALS-associated gene sets, then describe enrichment map explorations for each of the five categories representing common functions of the associated gene sets (immune response, developmental, nervous system, muscle, and lipid metabolism). High-resolution versions of the enrichment map figures are also provided (Figures S1–S6).

### 2.3. Mechanistic Relationships of ALS-Associated Gene Sets

The degree score (i.e., the number of neighboring nodes) for each of the 24 strongly associated gene sets is shown in Table 2. The CREB pathway is the most popular node of the enrichment map, having a degree of 50. The functional category having the largest number of gene sets in the enrichment map is the immune response, for which 7 gene sets pass FDR 0.05, 3 of which have low degrees and are not members of the main cluster.

In the following subsections, each functional category is explored separately, beginning with the immune system.

#### 2.3.1. Immune-Response Pathways

**Figure 3:**
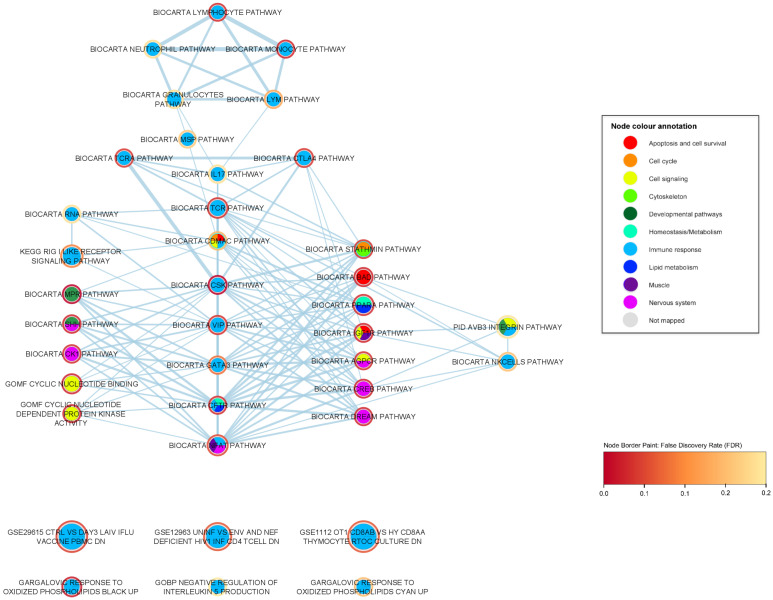
The immune response subnetwork, which includes 26 ALS-associated gene sets (FDR < 0.25, *p*-value < 0.05) related to immune response, along with their first significant neighbors (i.e., overlapping gene sets with FDR < 0.05). The size of each node is indicative of the number of genes within each gene set. The statistical significance of a gene set is represented by the intensity of its node’s red border, as shown in the legend at the right. Each connecting edge represents the proportion of genes shared between two gene sets, its width reflecting the size of this overlap. Color segments of each node represent one or more biological categories that we have classified as directly relevant from related literature, as shown in the legend at the right.

We report 26 significant gene sets that are related to the immune response. To further investigate these immune-response gene sets, we created a sub-network (shown in Figure 3) containing 38 nodes and 132 edges, including the 26 significant immune-response gene sets and first neighbours having statistical significance (FDR < 0.05), i.e., significant gene sets of any biological category that share a proportion of genes with any of the immune response gene sets. In this enrichment map network, an edge represents the overlapping genes that two gene sets share; the higher the width of an edge, the greater the overlap (edge similarity cut-off > 0.1). In Table 3, we summarize the immune-response related gene sets and their associated *p*-values, FDR values, degree, i.e., the number of edges of each node within the immune-response ALS enrichment network, and their corresponding number of genes.

It is noteworthy that the most popular immune response nodes (i.e., having the greater number of first neighbors) in this subnetwork are also the highest associated gene sets to ALS of the immune response category. The 8 most popular nodes (summarized in Table 3) include the BioCarta CSK pathway (*p*-value = 5.02 $\times \;10^{-5}$, FDR = 0.009), BioCarta CFTR pathway (*p*-value = 0.002, FDR = 0.046), BioCarta TCR pathway (*p*-value = 0.002, FDR = 0.048), BioCarta VIP pathway (*p*-value = 0.003, FDR = 0.049), BioCarta CTLA4 pathway (*p*-value = 0.004, FDR = 0.054), BioCarta NFAT pathway (*p*-value = 0.005, FDR = 0.07), BioCarta GATA3 pathway (*p*-value = 0.010, FDR = 0.099), and BioCarta CDMAC pathway (*p*-value = 0.024, FDR = 0.179). Several of these popular nodes and some non-popular nodes of the immune-response subnetwork are implicated in the T-cell receptor (TCR) signaling pathway (BioCarta TCR pathway), a key immune response mechanism, and concern lymphoid cell pathways, e.g., BioCarta TCR pathway, BioCarta CSK pathway, BioCarta VIP pathway, BioCarta TCRA pathway (*p*-value = 0.010, FDR = 0.099), BioCarta Lymphocyte pathway, BioCarta CTLA4 pathway, BioCarta NFAT pathway and BioCarta IL17 pathway (*p*-value = 4.31 $\times \;10^{-2}$, FDR = 0.22). Specifically, the highest ALS-associated gene set in our analysis BioCarta CSK pathway (FDR = 0.009) plays a role in the inhibition of T-cell receptor signaling and T-cell activation [24,25,32]. In the CSK pathway, the activated CSK kinase (COOH-terminal Srk kinase), which is transported to the plasma membrane through lipid rafts, phosphorylates the kinase Lck, which leads to the inhibition of the T-cell activation and T-cell signaling [24,25,32]. CTLA-4 (cytotoxic T-lymphocyte antigen-4) is a receptor expressed on the surface of T cells that leads to decreased T-lymphocyte activity and is considered a key immune response regulator [33]. The CTLA-4 pathway induces co-stimulatory signals during T-cell activation, providing additional control mechanisms that prevent inappropriate and hazardous T-cell activation that could lead to autoimmune disease pathogenesis [24,25,33]. In addition, the TCRA (T-cell receptor activation) pathway involves the activation of the T-Cell receptor through the Lck and Fyn tyrosine kinases [24,25]. Furthermore, the VIP (vasoactive intestinal peptide) pathway inhibits the apoptosis of activated T cells through two neuropeptides VIP and PACAP (pituitary adenylate cyclase-activating polypeptide) present in the lymphoid microenvironment, which have been known for their neuroprotective and immunomodulatory roles [24,25,34]. The BioCarta IL17 pathway (*p*-value = 4.31 $\times \;10^{-2}$, FDR = 0.22) concerns the secretion of the cytokine IL-17 by activated T cells as part of the inflammatory response and has been associated with autoimmune disorders [24,25]. NFAT is a transcriptional regulator that is also associated with the activation of the T cells, and plays a crucial role in the development and function of the immune system [35]. Lastly, the GATA-3 pathway is another popular node of the immune subnetwork. GATA-3 is a transcription factor which influences the development and differentiation of peripheral T cells, is specifically involved in the activation of the Th2 cytokine genes expression, and has been associated with allergic and lymphoproliferative disorders [24,25,36].

A number of the immune-response popular nodes and their first neighbors are associated with the innate/non-specific immune system, i.e., the first-line immune protection against foreign substances, viruses, bacteria, etc. These pathways include the BioCarta Monocyte pathway (*p*-value = 9.34 $\times \;10^{-4}$, FDR = 0.040), BioCarta CFTR pathway (*p*-value = 0.002, FDR = 0.046), KEGG RIG-I-like receptor signaling pathway (*p*-value = 0.002, FDR = 0.115), BioCarta CDMAC pathway (*p*-value = 0.024, FDR = 0.179), BioCarta NK cells pathway (*p*-value = 0.028, FDR = 0.190), BioCarta Neutrophil pathway (*p*-value = 0.040, FDR = 0.226), BioCarta Granulocytes pathway (*p*-value = 0.040, FDR = 0.226) and BioCarta RNA pathway (*p*-value = 0.042, FDR = 0.229). The monocyte pathway plays an important role in the innate immune response. Monocytes belong to the class of phagocytes. They can form macrophages or dendritic cells, and their role is to protect against bacterial, viral and fungal infections. The CFTR (cystic fibrosis transmembrane conductance regulator) protein is a chloride channel in the plasma membrane of epithelial cells, and certain CFTR mutations are the monogenic cause of cystic fibrosis [37]. The CFTR pathway has also been involved, among others, with the innate immune system and antimicrobial host defense, and the CFTR protein is expressed in macrophages and neutrophils [37,38]. The RIG-I-like (retinoic acid-inducible gene I) receptor signaling pathway involves the recognition of intracellular viral replication in the cells, as well as the initiation of inflammatory responses to eliminate the virus infection [39]. The aforementioned gene set includes, among others, the previously ALS-associated gene TBK1 (TANK binding kinase 1) with a statistical significance in our ALS gene-level analysis of FDR = 0.06. In addition, the CDMAC pathway, a popular node in our immune response subnetwork, stimulates the inappropriate proliferation and the DNA synthesis of the macrophages through the interaction of cadmium and G-protein coupled receptors [40]. Cadmium has been also associated with the inhibition of DNA repair and of the immune system, as well as activation of stress genes [40]. Neutrophils belong to the categories of granulocytes and phagocytes and play an essential role in the innate immune system and in inflammation. The natural killer cells pathway involves cytotoxicity mediated by natural killer cells, granular lymphocytes that are critical in the innate immune system. Lastly, the RNA pathway concerns the defense against viral infection by the PKR (protein kinase R), an interferon-induced protein kinase, which is activated by double-stranded RNA. Its anti-viral role mainly concerns blocking the viral translation mechanism and inducing apoptosis in the infected cells [41].

Several immune-response gene sets are linked to inflammatory regulation, a physiological part of the innate immune response, interleukins (ILs), a group of cytokines, and oxidative stress. Our results show a strong ALS association to oxidized phospholipids, the fourth most highly ALS-associated gene set of our analysis (and second in the immune-response category), Gargalovic response to oxidized phospholipids black up (*p*-value = 9.54 $\times \;10^{-6}$, FDR = 0.032), and the less significantly associated gene set, Gargalovic response to oxidized phospholipids cyan up (*p*-value = 2.38 $\times \;10^{-4}$, FDR = 0.187). Both of these gene sets derive from a study by Gargalovic et al. [42] of genes regulated by a specific oxidized phospholipid (the colors black and cyan refer to gene correlation clusters defined in that study). Oxidized phospholipids have been linked to pro-inflammatory and anti-inflammatory responses and atherogenesis through the stimulation of endothelial cells to produce inflammatory cytokines [24,25,42,43]. Furthermore, it has been shown that oxidized phospholipids are involved in acute and chronic microbial infections, metabolic disorders, and neurodegenerative diseases [43]. The BioCarta IL17 pathway (*p*-value = 4.31 $\times \;10^{-2}$, FDR = 0.22) concerns the secretion of the cytokine IL-17 by activated T cells, as part of the inflammatory response [24,25]. While the inflammatory cytokine IL-17 has been associated with pro-inflammatory properties, it has also been proven to play a critical role in autoimmune diseases, cancer progression and immunopathology [44,45]. The GOBP negative regulation of interleukin 5 production (*p*-value = 6.38 $\times \;10^{-4}$, FDR = 0.24) gene set is associated with the negative regulation of the cytokine IL-5, which is primarily known as a key mediator in the differentiation, growth, survival, and degranulation of eosinophils [46]. IL-5 is mainly produced by T helper-2 (Th2) lymphocytes, mainly involved in the response to parasites and allergies, and group 2 innate lymphoid cells (ILC2), and its expression is regulated by several transcription factors, such as GATA3 (BioCarta GATA3 pathway (*p*-value = 0.009, FDR = 0.099) [46,47]. Lastly, integrins—cell adhesion transmembrane receptors—(PID integrin cs pathway *p*-value = 0.005, FDR = 0.148; PID AVB3 integrin pathway *p*-value = 0.012034, FDR = 0.235) have been previously associated with cytokine activation as well as playing a critical role in infection, leukocyte recruitment, inflammation, angiogenesis and immunological signaling  [48].

#### 2.3.2. Developmental Pathways

**Figure 4:**
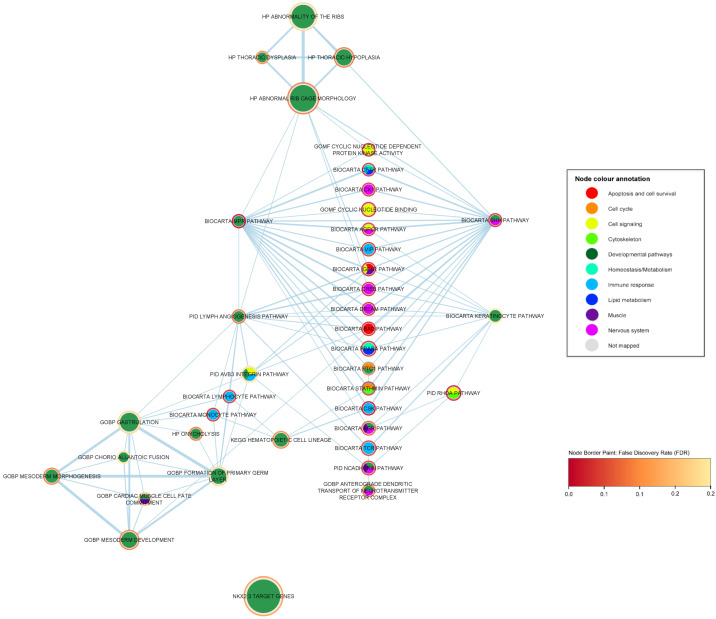
The developmental subnetwork that includes 22 ALS-associated gene sets (FDR < 0.25, *p*-value < 0.05) which are related to developmental processes, along with their first significant neighbors, i.e., associated gene sets (FDR < 0.05). The size of each node indicates the number of genes within each gene set. The statistical significance of each gene set/node is represented by the red color spectrum line outside each node (nodes with darker shades of red have a higher association with ALS, as shown on the legend at the right). The edge that connects two nodes represents the gene overlap between them, and the edge width reflects the amount of this particular overlap. Each node is colored by one or more biological categories that we have classified as directly relevant from related literature, as shown on the label at the right.

Another prominent biological category is developmental pathways. We report 22 ALS-associated gene sets that are related to human development (see Table 4). To investigate the significance of the developmental pathways in more depth, we constructed a developmental subnetwork (shown in Figure 4), containing 39 nodes and 101 edges, including the 22 significant developmental gene sets and their first neighbors, which show a statistically significant association to ALS (FDR < 0.05), i.e., significant gene sets of any biological category that share a number of genes with any of the developmental gene sets in distance one. In this enrichment map network, an edge represents the overlapping genes that two gene sets share; the higher the width of each edge, the larger the overlap (edge similarity cut-off > 0.1).

The BioCarta mPR (membrane progesterone receptor) pathway (*p*-value = 6.27 $\times \;10^{-5}$, FDR = 0.009), which involves the oocyte maturation by progesterone, is the highest ALS-associated gene set among the developmental-related gene sets, along with the immune-response BioCarta CSK pathway (*p*-value = 5.02 $\times \;10^{-5}$, FDR = 0.009), covered in the previous section. The BioCarta mPR pathway concerns the oocyte maturation by progesterone [24,25]. Progesterone is an endogenous steroid hormone that plays multiple roles, including oocyte meiotic maturation, embryogenesis, maintenance in pregnancy, and neural functions [49,50,51]. The binding of progesterone to intracellular and plasma membrane progesterone G protein-coupled receptors initiates a cascade of signaling pathways, including the indirect activation of the MAPK signaling. MPRs are G protein-coupled receptors (GPCRs) that have been previously associated with neuroprotective, neurosteroid and neuroendocrine functions in neurons [51]. The second most highly associated pathway in the developmental category is the Sonic Hedgehog (Shh) pathway (BioCarta Shh pathway *p*-value = 6.60 $\times \;10^{-4}$, FDR = 0.040). The Shh protein plays a critical role in the development processes in multi-cellular organisms through complex signaling cascades [52]. The Shh pathway has a key role in the cellular differentiation of multiple organs, in embryonic development, repair processes and especially in neuronal development [52,53]. Specifically, the Shh signaling pathway has been strongly associated, among others, with the development of the neural tube, motor neurons, the regulation of CNS polarity, neuronal regeneration and proliferation, stem cell renewal, and patterning of the developing thalamus and ventral forebrain [52,53,54]. Shh is also involved with proliferation-linked signaling cascades after binding to a receptor complex, including Ptc-1 and smoothed G-protein coupled receptor [24,25].

It is noteworthy that, in the developmental subnetwork, the two most highly associated developmental gene sets, BioCarta mPR and Shh pathways, are the most popular nodes but also share an almost complete overlap of statistically significant first neighbors (as shown in Figure 4 and Table 5). The most highly ALS-associated common neighbor is the immune-response BioCarta CSK pathway (*p*-value = 5.02 $\times \;10^{-5}$, FDR = 0.009) related to the inhibition of T-cell receptor signaling and T-cell activation [32]. Other immune-response common neighbors of the mPR and the Shh pathways include the BioCarta VIP pathway, which is also implicated in the T-cell signaling [34] and the BioCarta CFTR pathway, which has been associated among others with the innate immune system, B-cell activation and proliferation [37,38]. In addition, we observe cell signaling pathways as common neighbors for the two developmental gene sets, such as the GOMF cyclic nucleotide dependent protein kinase activity, GOMF cyclic nucleotide binding, BioCarta IGF1R pathway and the BioCarta AGPCR pathway. Cyclic nucleotides are secondary messengers that play a central role in intracellular signal transduction, responding to hormonal stimuli and intra- or extracellular environmental changes [55,56]. The BioCarta AGPCR pathway relates to the signaling attenuation of the G-protein coupled receptors (GPCR) [24,25], a transmembrane protein family which is well known for its major role in the signal transduction of extracellular stimuli across the plasma membranes. Failure to attenuate the rapid GPCR signaling leads to acute and chronic overstimulation of the receptors [57]. The IGF-1R pathway involves multiple anti-apoptotic pathways through the IGF-1R (type 1 receptor for insulin-like growth factor), promoting cell survival and growth, as well as blocking apoptotic pathways such as the BioCarta BAD pathway, another statistically significant common neighbor. Furthermore, we note the presence of several nervous system-specific signaling pathways, including the BioCarta DREAM pathway, BioCarta CREB pathway, BioCarta CK1 pathway and BioCarta AGPCR pathway gene sets. The DREAM (downstream regulatory element antagonistic modulator) pathway involves the repression of the pain sensation by the DREAM transcriptional regulator [58]. DREAM has been linked to pain signaling and is expressed in spinal cord neurons [58]. The CREB (cyclic AMP-responsive element binding) pathway mediates the activation of transcription in response to extracellular stimuli including neurotransmitters, hormones, membrane depolarization, and growth and neurotrophic factors, by the transcription factor CREB [59]. The CK1 (casein kinase 1) pathway includes the dopaminergic signaling in the neostriatum, elevating cAMP (a type of cyclic nucleotide) and activating PKA (protein kinase A) [24,25]. It has been argued that CK1 family members are signal transduction regulators in the Shh and Wnt developmental signaling pathways [60]. Lastly, we observe the presence of gene sets relating to lipid metabolism and homeostasis, such as the BioCarta CFTR pathway and the BioCarta PPAR-alpha pathway. The PPAR-alpha (peroxisome proliferator activated receptor alpha) pathway relates to the gene regulation by peroxisome proliferators via the PPAR-alpha phosphoprotein and has been linked to fatty acid metabolism regulation, and autophagy in human microglia and hepatic cells [61].

The third most highly associated gene set in the developmental pathways category concerns the signaling pathways induced by N-cadherin (PID N-cadherin pathway *p*-value = 5.77 $\times \;10^{-4}$, FDR = 0.057) (shown in Table 4). Neural cadherin (N-cadherin) is an adhesion receptor mainly known for its key role in the organization of the synaptic complex, ensuring the adhesion between synaptic membranes and organizing the actin cytoskeleton, as well as being involved in cell type specific adhesion processes during embryonic development [62]. N-cadherin is ubiquitously expressed in the neuronal synapses and in the vascular smooth muscle, participating in the development and plasticity of the adult neural tissue [63,64,65]. N-cadherin has also been associated with the mediating of signal transduction events during bone development and vasomotor control [66,67]. The PID N-Cadherin pathway gene set is connected with the ALS-associated gene set PID RhoA pathway (*p*-value = 1.16 $\times \;10^{-4}$, FDR = 0.022). Ras homolog gene family member A (RhoA) is a small GTPase that has an essential role in regulating the development, differentiation, survival, and death of neurons in the central nervous system [68]. The link between N-cadherin and RhoA has been previously reported by other studies, highlighting the cell signaling capabilities of N-cadherin modulating the voltage activated calcium influx by RhoA activity mechanisms and its downstream effects on the cytoskeleton [69]. In addition, we observe that the N-cadherin pathway is linked to the immune-response T-cell receptor signaling pathway gene set BioCarta TCR pathway (*p*-value = 0.002, FDR = 0.048). Furthermore, in the developmental subnetwork, we observe the N-cadherin gene set to be connected to the GOBP anterograde dendritic transport of neurotransmitter receptor complex (*p*-value = 5.60 $\times \;10^{-5}$, FDR = 0.110), which relates to the directed neurotransmitter receptor complex movement toward the post-synapse through dendritic transport [24,25]. The PID AVB3 integrin pathway (*p*-value = 0.012, FDR = 0.236) is another immediate neighbor of the N-cadherin pathway. Integrins, such as cadherins, are well-studied cell adhesion molecules that mediate cell–cell and/or cell–extracellular matrix (ECM) adhesion, involved in several signaling pathways, and immune-response processes as well as having developmental roles. The PID AVB3 integrin pathway represents specifically the role of the integrins in angiogenesis [24,25].

We further observe two highly interconnected clusters in the developmental subnetwork. The first concerns pathways related to abnormalities in the morphology of the rib cage/thorax, including the gene sets HP thoracic hypoplasia (*p*-value = 5.01 $\times \;10^{-5}$, FDR = 0.101), HP abnormal rib cage morphology (*p*-value = 6.03 $\times \;10^{-5}$, FDR = 0.101), HP thoracic dysplasia (*p*-value = 9.63 $\times \;10^{-5}$, FDR = 0.122) and HP abnormality of the ribs (*p*-value = 2.65 $\times \;10^{-4}$, FDR = 0.223). All four gene sets share the previously associated ALS gene NEK1 (NIMA-related kinase 1) (*p*-value = 0.007, FDR = 0.27). In addition, we note a second cluster which is linked with gastrulation, an early embryonic developmental process of the blastocyst forming a multilayer gastrula, including the endoderm, mesoderm, and ectoderm (GOBP gastrulation; *p*-value = 5.13 $\times \;10^{-4}$, FDR = 0.218; GOBP Formation of primary germ layer *p*-value = 4.68 $\times \;10^{-4}$, FDR = 0.218) [70]. Gastrulation is also associated with embryonic placenta development, including cell–cell adhesion processes, such as chorioallantoic fusion (GOBP chorio allantoic fusion *p*-value = 7.28 $\times \;10^{-4}$, FDR = 0.242) as well as the formation and the morphogenesis of anatomical structures that derive from the mesoderm—the second germ layer that develops, among others, into smooth, cardiac, and skeletal muscle, bone, reproductive organs, microglia, adrenal cortex, cartilage, blood cells, vascular endothelium and connective tissue (GOBP mesoderm morphogenesis *p*-value = 1.65 $\times \;10^{-5}$, FDR = 0.111; GOBP mesoderm development *p*-value = 7.52 $\times \;10^{-5}$, FDR = 0.111; GOBP cardiac muscle cell fate commitment *p*-value = 1.99 $\times \;10^{-4}$; FDR = 0.190) [70]. We further observe multiple links of the gastrulation-related gene sets to statistically significant immune-response gene sets (i.e., BioCarta Monocyte pathway, BioCarta lymphocyte pathway, and PID AVB3 integrin pathway).

We note the central role of the developmental gene set PID lymph angiogenesis pathway (*p*-value = 7.28 $\times \;10^{-4}$, FDR = 0.095) (shown in Figure 4) referring to the VEGFR3 (Vascular Endothelial Growth Factor Receptor 3) signaling in the lymphatic endothelium [24,25,71]. The lymph angiogenesis pathway shares many of the common neighbors of the mPR and the Shh top developmental pathways, including the nervous system-specific cell-signaling pathways, BioCarta IGF1R pathway, BioCarta DREAM pathway, BioCarta CREB pathway, apoptotic BioCarta BAD pathway and BioCarta PPAR-alpha pathway which is linked to lipid metabolism and homeostasis. In addition, the lymph angiogenesis gene set is a bridging node between the two distinct interconnected cliques that were described in the previous subsubsection, the one relating to rib cage dysplasias, and the second one including gene sets linked to gastrulation and mesoderm morphogenesis. Lastly, the PID lymph angiogenesis pathway is linked to several immune-response significant gene sets, such as the BioCarta lymphocyte, monocyte and TCR pathways.

#### 2.3.3. Nervous System Pathways

**Figure 5:**
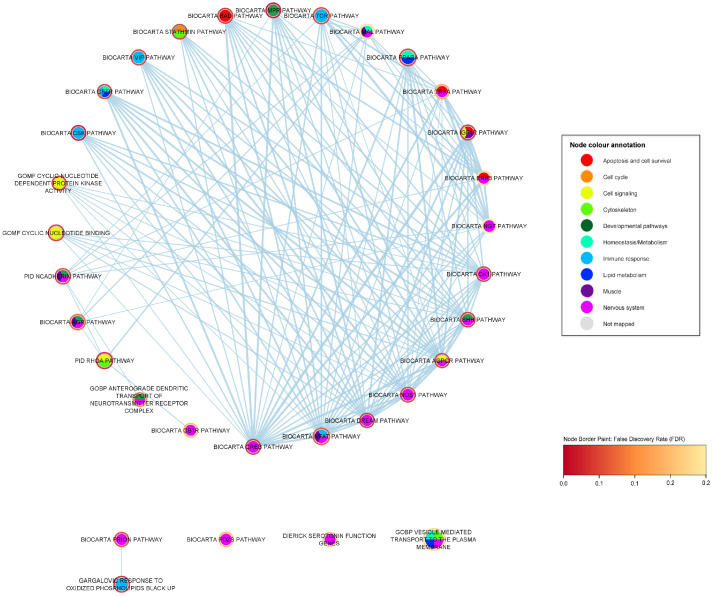
The nervous system subnetwork that includes 19 ALS-associated gene sets (FDR < 0.25, *p*-value < 0.05) which are related to nervous system processes along with their 13 first significant neighbors, i.e., ALS-associated gene sets (FDR < 0.05). The nervous system subnetwork is highly dense, and the nodes are circularly arranged by their degree in descending order. The size of each node indicates the number of genes within each gene set. The statistical significance of each gene set/node is represented by the red color spectrum line outside each node (nodes with darker shades of red have a higher association to ALS), as shown on the legend at the right. The edge that connects two nodes represents the gene overlap between them, and the edge width reflects the amount of this particular overlap. Each node is colored by one or more biological categories that we have classified as directly relevant from related literature, displayed in the legend at the right.

In this subsection, we focus on nervous system-specific pathways that are associated with ALS based on the results of our genome-wide gene-set analysis. We report 19 ALS-associated gene sets that have been known to be involved in nervous system pathways and biological processes (shown in Table 6). The nervous system-specific subnetwork contains, in total, 32 nodes and 142 edges, including 19 ALS-associated nervous system related gene sets and their significant first neighbors (with FDR < 0.05) which is shown in Figure 5.

The seven most popular ALS-associated gene sets within the nervous system subnetwork (with a ranging degree from 16 to 21) form an interconnected cluster (clique) (shown in Table 6). This popular clique includes the BioCarta CREB pathway (*p*-value = 4.05 $\times \;10^{-4}$, FDR = 0.039), BioCarta DREAM pathway (*p*-value = 0.001103, FDR = 0.040), BioCarta Shh pathway (*p*-value = 6.6 $\times \;10^{-4}$, FDR = 0.040), BioCarta CK1 pathway (*p*-value = 0.001, FDR = 0.046), BioCarta AGPCR pathway (*p*-value = 0.002, FDR = 0.048), BioCarta NOS1 pathway (*p*-value = 0.003, FDR = 0.055) and BioCarta NFAT pathway (*p*-value = 0.005, FDR = 0.068). The CREB (cyclic AMP response element-binding protein 1) is a stimulus-induced transcription factor that mediates the activation of transcription in response to extracellular stimuli. Stimulus-induced phosphorylation of CREB affects a variety of signaling cascades, including the ERK1/2, MAPK, PI3K/AKT, CaMK, PKC, and PKA [59]. The DREAM (downstream regulatory element antagonistic modulator) transcriptional regulator is expressed in spinal cord neurons and is involved in pain signaling [58]. The Sonic Hedgehog (Shh) pathway, the second most highly associated pathway in the developmental category, comes up as the third top gene set in the nervous system category as well. As mentioned in the developmental subsection, the Shh pathway plays a key role in neuronal development, among others [52,53]. Specifically, the Shh signaling pathway has been strongly associated among others with the development of the neural tube, differentiation of the floor plate cells, motor neurons, and inter-neurons, the regulation of CNS polarity, neuronal regeneration and proliferation, stem cell renewal, survival of ventral progenitors, specification of ventral neurons, midbrain dopaminergic differentiation, proliferative signaling cascades in the developing cerebellum and other tissues and patterning of the developing thalamus and ventral forebrain [52,53,54]. The CK1 (casein kinase 1) pathway includes dopaminergic signaling in the neostriatum through the activation of a G-protein coupled dopamine receptor by dopamine, elevating cAMP and activating PKA (protein kinase A) [24,25]. Protein kinase CK1 has also been associated with the glutamatergic synaptic transmission regulation mediated by N-methyl-D-aspartic acid (NMDA) receptors as well as with the signal transduction regulation in the Shh and Wnt developmental signaling pathways [60,72]. The AGPCR pathway refers to the signaling attenuation of the G-protein coupled receptors (GPCR), avoiding acute and chronic overstimulation of the receptors [24,25,57]. The NOS1 pathway concerns the glutamatergic-mediated nitric oxide (NO) production mediated by the NMDA postsynaptic density protein 95 (PSD95)-neuronal nitric oxide synthase (nNOS1) complex [24,25]. NO has an important role in inflammation through the up-regulation of NOS in microglia as well as in cardiovascular, reproductive, neuromuscular and nervous system functions [73]. Finally, the NFAT (nuclear factor of activated T cells) BioCarta pathway relates to hypertrophy of the cardiac muscle, but also plays a key role in the immune system, development of the nervous system and skeletal muscle [24,25,35]. The NFAT transcription factor has been also associated with the regulation of pro-inflammatory responses in cultured murine microglia [74].

The highly associated nervous system-related clique is also interconnected with six statistically significant gene sets that form links solely with this particular clique. These gene sets include immune response (i.e., BioCarta CSK pathway, BioCarta CFTR pathway and BioCarta VIP pathway), cell signaling (i.e., GOMF cyclic nucleotide-dependent protein kinase activity, and GOMF cyclic nucleotide binding) and cell cycle and cytoskeleton pathways (i.e., BioCarta Stathmin pathway). The majority of the former gene sets belong to the top seven ALS-associated gene sets of our analysis. Furthermore, the nervous system clique is connected to more popular nodes, including developmental pathways, such as the top gene set BioCarta mPR pathway, apoptotic gene sets, such as the BioCarta BAD pathway and the BioCarta IGF1R pathway (which is also closely associated with cell signaling and muscle-related processes), pathways related to homeostasis and lipid metabolism, such as the BioCarta PPAR-alpha pathway, and immune response-related gene sets, such as the BioCarta TCR pathway.

The BioCarta Prion pathway (*p*-value = 0.003, FDR = 0.054) is the sixth most highly ALS-associated gene set within the nervous system response-related category (shown in Table 6). The ALS-associated Gargalovic response to oxidized phospholipids black up (*p*-value = 9.54 $\times \;10^{-6}$, FDR = 0.032) gene set, which is related to immune response and oxidative stress, is linked with the prion pathway, which has been associated with neurotoxicity and neurodegeneration [75,76].

#### 2.3.4. Muscle Pathways

**Figure 6:**
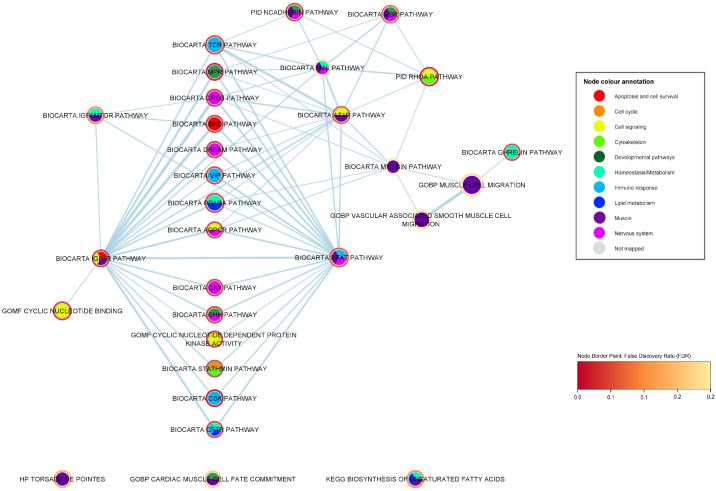
The muscle subnetwork includes 13 ALS-associated gene sets (FDR < 0.25, *p*-value < 0.05) which are related to muscle system processes along with their first significant neighbors, i.e., ALS-associated gene sets (FDR < 0.05). The size of each node indicates the number of genes within each gene set. The statistical significance of each gene set/node is represented by the red color spectrum line outside each node (nodes with darker shades of red have a higher association with ALS), shown on the legend at the right. The edge that connects two nodes represents the gene overlap between them, and the edge width reflects the amount of this particular overlap. Each node is colored by one or more biological categories that we have classified as directly relevant from related literature, displayed in the legend at the right.

Several of the ALS-associated processes and pathways could be of particular relevance to muscle cell functions. We report 13 ALS-associated gene sets that have been known to be involved in muscle pathways and biological processes (shown in Table 7). The muscle-specific subnetwork contains in total 30 nodes and 68 edges, including the 13 ALS-associated muscle system-related gene sets and their significant first neighbors (with FDR < 0.05), shown in Figure 6.

Several of the ALS-associated processes and pathways could be of particular relevance to muscle cell functions (shown in Table 7). These include a number of processes that are quite specific to muscle, such as agrin-mediated organization of the skeletal muscle cytoskeleton (BioCarta AGR pathway *p*-value = 0.007, FDR = 0.088), the AKT/mTOR pathway (BioCarta IGF1/mTOR pathway *p*-value = 0.012, FDR = 0.109) that has a key role in regulation of skeletal muscle mass, cardiac muscle cell fate commitment (GOBP cardiac muscle cell fate commitment *p*-value = 1.99 $\times \;10^{-4}$, FDR = 0.190), and muscle cell migration (GOBP muscle cell migration *p*-value = 4.75 $\times \;10^{-4}$, FDR = 0.218), as well as processes that are of particular importance to muscle but also have more broad roles in other tissues. Among the latter are: the phosphorylation of myosin (BioCarta Myosin pathway *p*-value = 0.048, FDR = 0.246), which is known to regulate smooth muscle contraction and platelet release; serum response factor-mediated transcriptional regulation of genes involved in the actin cytoskeleton and cell adhesion [77], including in cardiac and smooth muscle [78], as well as several other molecular pathways described in BioCarta or in the pathway interaction database—notably, due to their high significance, pathways of the IGF1-receptor (BioCarta IGF1R pathway *p*-value = 9.71 $\times \;10^{-4}$, FDR = 0.040), N-Cadherin (PID N-Cadherin pathway *p*-value = 5.77 $\times \;10^{-4}$, FDR = 0.057), NFAT (BioCarta NFAT pathway *p*-value = 0.005, FDR = 0.069), and the angiotensin receptor (BioCarta AT1R pathway *p*-value = 0.007, FDR = 0.077). The IGF1 pathway has a broad role in many tissues, including the induction of growth or differentiation of target cells, cell survival and maintenance of cell function, and is notable in muscle for the regulation of muscle mass [79]. The BioCarta IGF1R pathway involves multiple antiapoptotic pathways from IGF-1R signaling, leading to BAD phosphorylation [24,25]. N-cadherin is a cell–cell adhesion glycoprotein required for left–right asymmetry during gastrulation, with roles in the central nervous system, and in cardiac and skeletal muscle development [80]. The BioCarta NFAT pathway specifically relates to hypertrophy of the cardiac muscle, but includes all four members of the NFAT transcription factors that are important to the immune system and to the development of the nervous system and skeletal muscle, including myoblast fusion [81]. The AT1R pathway relates to the angiotensin receptor, and angiotensin II mediated activation [24,25]. Angiotensin II regulates many aspects of the cardiac muscle [82].

Most of these muscle-related gene sets overlap to a limited extent with one or more of the others, in terms of sharing genes in common, and most of the shared genes relate to core cellular signaling cascades (shown in Figure 6). There are only minor differences in gene membership between the muscle cell migration and vascular-associated smooth muscle cell migration gene sets. The BioCarta NFAT pathway and BioCarta IGF1R pathway gene sets have 42% similarity, both making use of the Ras/Raf/MEK/ERK signaling cascade. The BioCarta AKT/mTOR, AT1R, and MAL pathways each overlap both NFAT and IGF1R, again due to all of these pathways sharing the Ras/Raf/MEK/ERK signaling cascade. The phosphorylation of myosin and the angiotensin pathway both feature protein kinase C and the G protein subunit alpha. Certain G protein components as well as Rho-associated coiled-coil containing protein kinase 1 (ROCK1) are in common between myosin phosphorylation and muscle cell migration. The BioCarta AT1R pathway and BioCarta MAL pathway share not only the Ras/Raf/MEK/ERK signaling cascade, but other signaling components such as RAC1 and MAPK8 (JNK). ERK, RAC1, and JNK are each also core components of the AGR pathway, explaining its overlap with the MAL and ATR1 pathways. The BioCarta MAL pathway and the PID N-Cadherin pathway share some of these same signaling components, including JNK, ROCK1, RhoA, and RAC1.

A number of other gene sets that have robustly significant (FDR < 0.05) genome-wide association to ALS overlap with these muscle-related gene sets, the greatest overlap being with the BioCarta NFAT pathway and BioCarta IGF1R pathway (shown in Figure 6). Core signaling cascades are again prominent among the shared genes. For example, the NFAT and IGF1R pathways each share the following: cAMP-dependent protein kinases and some calmodulin signaling with the Stathmin pathway; protein kinases with the VIP pathway (also calmodulin signaling, in the case of NFAT); several protein kinases and phosphatases with the CK1 pathway; protein kinases, AKT1, and ERK1, with the CREB pathway (along with much more in the case of IGF1R, the CREB and IGF1R pathways having 63% overlap); and numerous protein kinases with the AGPCR pathway. The BAD pathway overlaps with the NFAT and the AKT/mTOR pathways, sharing components of each, while the AKT/mTOR pathway also overlaps with the CREB pathway. IGF1 and IGF binding proteins 3 and 5 are shared by muscle cell migration and the ghrelin pathway. Components (though not always the same components) of the PID RhoA pathway are shared by the N-Cadherin, AT1R, AGR, MAL, and myosin pathways.

#### 2.3.5. Lipid Metabolism Pathways

**Figure 7:**
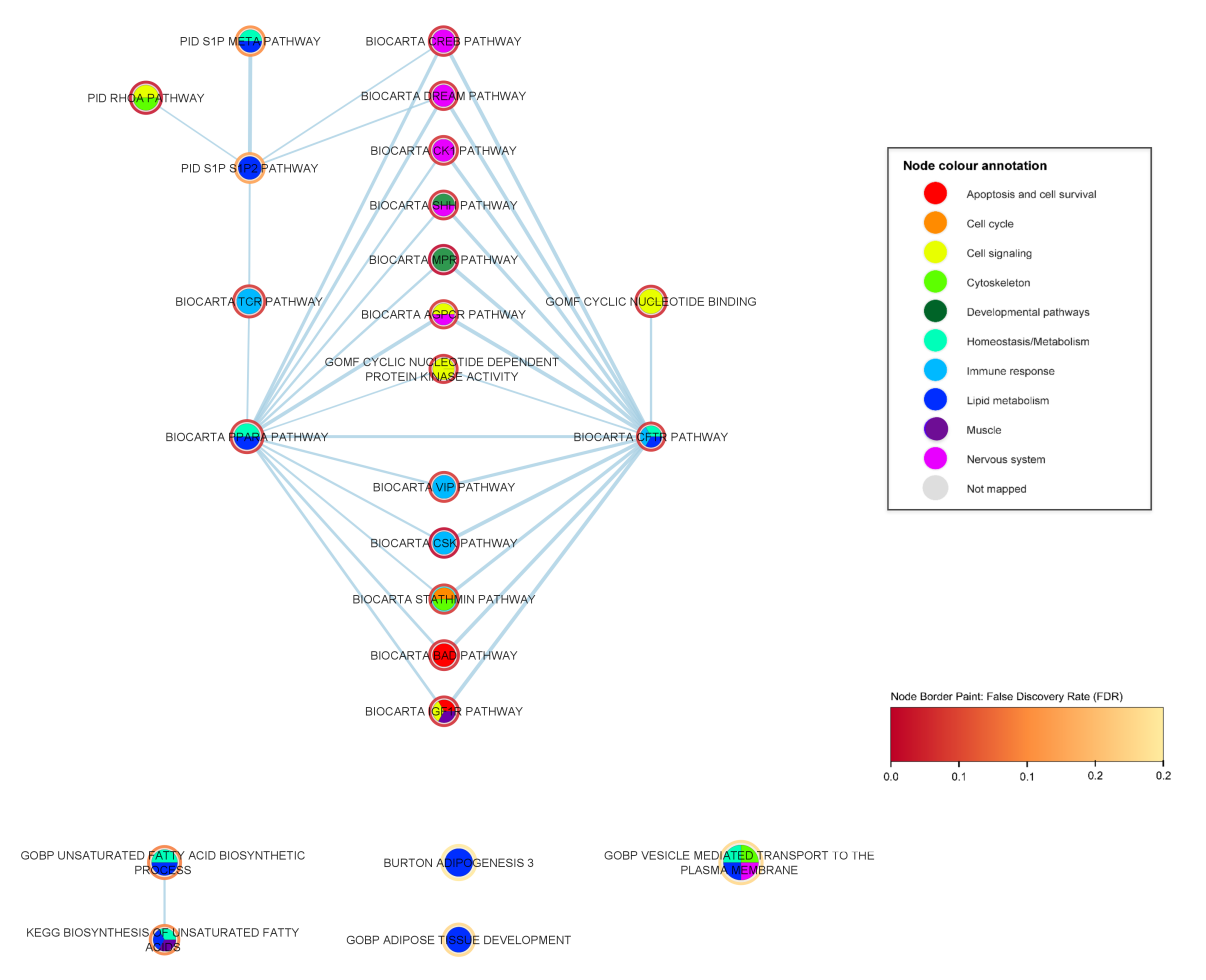
The lipid metabolism subnetwork that includes 9 ALS-associated gene sets that are related to lipid metabolism processes (FDR < 0.25, *p*-value < 0.05) along with their 15 first significant neighbors, i.e., ALS-associated gene sets (FDR < 0.05). The size of each node indicates the number of genes within each gene set. The statistical significance of each gene set/node is represented by the red colour spectrum line outside each node (nodes with darker shades of red have a higher association with ALS), as shown on the legend at the right. The edge that connects two nodes represents the gene overlap between them, and the edge width reflects the amount of this particular overlap. Each node is colored by one or more biological categories that we have classified as directly relevant from related literature, as shown on the legend at the right.

We report 9 ALS-associated gene sets that are related to lipid metabolism (shown in Table 8). The lipid metabolism subnetwork is a small network compared to the previous subnetworks, containing, in total, 24 nodes and 33 edges, including the 9 ALS-associated lipid metabolism-related gene sets and their significant first neighbors (with FDR < 0.05) as shown in Figure 7.

The BioCarta PPAR-alpha pathway (*p*-value = 0.002, FDR = 0.046) and BioCarta CFTR pathway (*p*-value = 0.002, FDR = 0.046) are the top two gene sets within the lipid metabolism category but also the most popular nodes within the lipid metabolism subnetwork, with a common degree of 14 (shown in Table 8). Both gene sets are related to lipid metabolism but also to homeostasis/cell metabolism processes, while the CFTR pathway is also linked with immune response processes. Specifically, the CFTR (cystic fibrosis transmembrane conductance regulator) protein is a chloride channel in the plasma membrane of epithelial cells, and certain CFTR mutations are the monogenic cause of cystic fibrosis [37]. The CFTR pathway has also been involved among others with the innate immune system and antimicrobial host defence [37,38]. Furthermore, the BioCarta PPAR-alpha (peroxisome proliferator activated receptor alpha) pathway relates to the gene regulation by peroxisome proliferators via the PPAR-alpha phosphoprotein [24,25]. Peroxisomes are subcellular metabolic organelles found in almost all eukaryotic cells that play a key role in lipid metabolism and redox homeostasis, ensuring the proper cellular response to endogenous and exogenous stimuli [83,84]. The peroxisome proliferator-activated receptors (PPARs) belong to the superfamily of nuclear hormone receptors. PPAR-alpha, a PPAR isoform, affects the expression of target genes involved in cell proliferation, cell differentiation, immune and inflammation responses [61,85]. Specifically, PPAR-alpha has been established as a major regulator of the fatty acid metabolism in hepatic cells and has also been linked to autophagy in human microglia, oxidative phosphorylation and regulation of energy homeostasis cells [61,85]. PPAR-alpha expression is ubiquitous with high expression levels in tissues with a high level of fatty acid catabolism, such as the liver, heart, and muscle, but has been also detected in immune cells and specifically in T cells and macrophages [85,86]. Lastly, PPARs have been previously associated with the regulation of the oxidative metabolism of skeletal muscle and the promotion of fiber-type conversion by mediating the activity of peroxisome proliferator [87].

The vast majority of the first neighbors (12 nodes out of 14) of the two popular lipid-related gene sets BioCarta PPAR-alpha pathway and BioCarta CFTR pathway, are mutually shared, as shown in Figure 7. The most frequent biological category among the commonly shared first neighbors (FDR < 0.05) is related to nervous system pathways and processes (i.e., BioCarta CREB pathway, BioCarta Shh pathway, BioCarta DREAM pathway, BioCarta CK1 pathway and BioCarta AGPCR pathway). The rest of the biological categories concern developmental gene sets (i.e., BioCarta Shh pathway and BioCarta mPR pathway), cell signaling gene sets (i.e., BioCarta AGPCR pathway, GOMF Cyclic nucleotide dependent protein kinase activity and BioCarta IGF1R pathway) and immune response gene sets mostly related to T-cell activation (i.e., BioCarta CSK pathway BioCarta VIP pathway). Lastly, the BioCarta TCR pathway, a significant T-cell receptor immune-response node, is solely linked to the BioCarta PPAR-alpha pathway gene set.

Apart from the 12 mutual neighbors of these two top lipid metabolism gene sets, the two gene sets also overlap (combined overlap similarity = 0.33), sharing 6 genes in total, including the *PRKACG*, *PRKAR1A*, *PRKAR2B*, *PRKACB*, *PRKAR1B* and *PRKAR2A*. The genes shared in common by BioCarta CFTR and PPAR-alpha pathways are protein-coding genes that are members of the serine/threonine protein kinase family, coding various subunits of the cyclic AMP (cAMP) dependent protein kinase, important to processes such as cell proliferation and differentiation [32]. Of these 6 shared genes, only *PRKACG* (protein kinase cAMP-activated catalytic subunit gamma) reaches statistical significance (FDR = 0.129).

### 2.4. Interaction Analysis

We performed interaction gene-set analysis, using MAGMA (v1.10), across all 145 gene sets found in our earlier analysis with FDR < 0.25, aiming to investigate how the genes shared between pairs of gene sets (interaction term) may contribute to the significance of individual gene sets, i.e., whether the statistical significance of two sets may be derived from the genes shared between them.

Upon running interaction analysis, MAGMA yielded 71 valid interactions between all possible pairs of gene sets in the analysis, of which 4 reached marginal statistical significance (*p*-value < 0.05), shown in Table 9.

To interpret the interaction results, Enrichr [88,89,90] was used to investigate the functional enrichment in gene ontology terms of the shared genes between the interacting gene sets. The interactions of the BioCarta PPAR-alpha and GPCR pathways, as well as the BioCarta PPAR-alpha and CREB pathways, yielded the same top enriched gene sets (sorted by their adjusted *p*-values using the Benjamini–Hochberg multiple testing correction method) in gene ontology biological processes (BP), molecular functions (MF) and cellular components (CC), including activation of protein kinase A activity (GOBP:0034199) (PPAR-alpha ∩ GPCR *p*-value = 2.92 $\times \;10^{-17}$, q-value = 7.50 $\times \;10^{-15}$; PPAR-alpha ∩ CREB *p*-value = 1.28 $\times \;10^{-16}$, q-value = 4.20 $\times \;10^{-14}$), cAMP-dependent protein kinase inhibitor activity (GOMF:0004862) (PPAR-alpha ∩ GPCR *p*-value = 2.20 $\times \;10^{-12}$, q-value = 9.47 $\times \;10^{-11}$; PPAR-alpha ∩ CREB *p*-value = 5.19 $\times \;10^{-12}$, q-value = 2.34 $\times \;10^{-10}$) and plasma membrane raft (GOCC:0044853) (PPAR-alpha ∩ GPCR *p*-value = 7.80 $\times \;10^{-6}$, q-value = 1.24 $\times \;10^{-4}$; PPAR-alpha ∩ CREB *p*-value = 1.27 $\times \;10^{-7}$, q-value = 3.04 $\times \;10^{-5}$). In addition, the shared genes by the KEGG Hematopoietic cell lineage and ECM receptor interaction were enriched in: extracellular structure organization (GOBP:0043062) (*p*-value = 3.23 $\times \;10^{-15}$, q-value = 4.75 $\times \;10^{-13}$), external encapsulating structure organization (GOBP:0045229) (*p*-value = 3.37 $\times \;10^{-15}$, q-value = 4.75 $\times \;10^{-13}$), collagen binding involved in cell-matrix adhesion (GOMF:0098639) (*p*-value = 4.54 $\times \;10^{-5}$, q-value = 4.09 $\times \;10^{-4}$), neuregulin binding (GOMF:0038132) (*p*-value = 4.54 $\times \;10^{-5}$, q-value = 4.09 $\times \;10^{-4}$) and focal adhesion (GOCC:0005925) (*p*-value = 4.96 $\times \;10^{-11}$, q-value = 1.00 $\times \;10^{-9}$). Lastly, the top ontology enrichment results for the genes shared by the KEGG Gap junction and the BioCarta GPCR pathway, are activation of protein kinase A activity (GOBP:0034199) (*p*-value = 6.10 $\times \;10^{-8}$, q-value = 1.27 $\times \;10^{-5}$), protein serine/threonine kinase activity (GOMF:0004674) (*p*-value = 3.43 $\times \;10^{-7}$, q-value = 1.24 $\times \;10^{-5}$), late endosome (GOCC:0005770) (*p*-value = 0.004, q-value = 0.079) and early endosome (GOCC:0005769) (*p*-value = 0.007, q-value = 0.079).

## 3. Discussion

**Figure 8:**
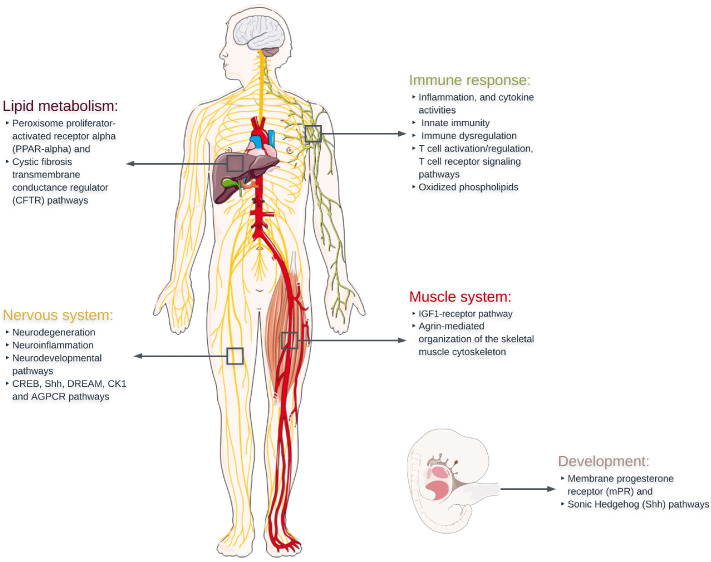
Overview of the main ALS pathways identified by the genome-wide gene-set analysis. The main affected ALS mechanisms are categorised in: (1) immune response processes (green) such as inflammation, cytokine activities, innate immunity, immune dysregulation, T-cell activation/regulation, T-cell receptor signaling pathways and oxidized phospholipids; (2) lipid metabolism pathways (purple) including the top peroxisome proliferator-activated receptor alpha (PPAR-alpha) and cystic fibrosis transmembrane conductance regulator (CFTR) gene-sets; (3) muscle system gene sets (red) such as the highest associated IGF1-receptor and agrin-mediated organization of the skeletal muscle cytoskeleton pathway; (4) nervous system processes (yellow) related to neurodegeneration, neuroinflammation, neurodevelopment and Cyclic AMP response element-binding protein 1 (CREB), Sonic Hedgehog (Shh), downstream regulatory element antagonistic modulator (DREAM), casein kinase 1 (CK1) and signaling attenuation of the G-protein coupled receptors (AGPCR) pathways; and (5) developmental pathways (light brown) with top gene sets, the membrane progesterone receptor (mPR) and Sonic Hedgehog (Shh) pathways.

This study has identified genes and functional gene sets that are associated with amyotrophic lateral sclerosis, and the functions of these gene sets have been explored using network approaches aiming to understand the underlying pathology of the disease. Twenty-four gene sets were observed to have robust (FDR < 0.05) association to ALS. The functional roles of these gene sets mainly concerned neuron and embryonic development, the immune response, lipid metabolism, and nervous and muscle system processes (Table 2 and Figure 2). These main findings are also summarized in Figure 8.

### 3.1. Gene Level Confirmation

The MAGMA multi-model gene-level results of the ALS-control cohort yielded 6 genes that reached high statistical significance based on false-discovery rate (FDR < 0.01) and 4 genes that passed the stricter multiple testing correction method of Bonferroni (alpha = 0.05; *p*-value < 2.58 $\times \;10^{-6}$). The reported genes have been previously associated with ALS by numerous studies, supporting the reproducibility of the present analysis. Some of the more well-established ALS-associated genes in our analysis include *C9ORF72* [30], *UNC13A* [31] and *KIF5A* [9], which were also reported in the original GWAS study reporting the ALS cohort that we used [9]. The analysis also revealed two previously identified ALS-associated genes *TBK1* (TANK binding kinase) (FDR = 0.063) and *FUS* (fused in sarcoma; FDR = 0.155) [31], with marginal statistical significance.

### 3.2. Gene Set Association

To interpret the gene set results a combination of manual curation, Cytoscape, and Enrichment Mapping, was used to visualize biological category-specific subnetworks. It is noteworthy that the majority (67%) of the 24 gene sets having robust (FDR < 0.05) association to ALS have also a very high degree (i.e., number of undirected edges shared with other gene sets/nodes) within the ALS enrichment network. These gene sets also form a highly interconnected cluster and are present in every subnetwork category, usually as one of the more popular nodes. An example of this concerns the BioCarta PPAR-alpha and CFTR gene sets within the lipid metabolism network, which are simultaneously the most popular nodes and the most highly associated with ALS within the lipid metabolism category. Gene sets/nodes with a high degree (also termed as hub nodes) are often of high functional relevance, indicating an important topological role in functionally connecting multiple biological pathways within a complex biological network [91]. This high connectivity and popularity among the most ALS-associated gene sets, which also expands to the rest of the subnetworks, may suggest important underlying roles of these particular pathways in ALS pathology. Among the most significant GSA results, pathways related to neuronal and immune systems were the most abundant, with relatively larger network size (in terms of nodes) and density (in terms of the number of edges) in comparison to other categories.

A driving rationale in this study was to include a general view of ALS mechanisms to enable the identification of overlapping functions while being as data-driven as possible. To satisfy this, our analysis tested some 31,454 gene sets, representing a huge range of functions, both specific but also quite generic, and thereby identifying a large number of gene set associations, which then presented a challenge for interpretation. Several of the identified gene sets represent processes or structures that are known or thought to be involved in secondary/downstream cellular pathology of ALS, including apoptosis, the cytoskeleton, homeostasis, the cell cycle, and immune response. However, care should be taken in interpreting these observed associations: the direction of causal relationship for a genomic association can only be from the genome to the disease, not the reverse. The mechanistic consequence of a genetic variant may indeed be downstream of other molecular-cellular events: in this case, certain characteristics (environmental or other genetic factors) might predispose an individual to ALS, but some of the identified disease-associated genomic features could be causal on top of (i.e., having downstream consequences of) those characteristics. The potential causal involvement of the identified gene sets, especially of pathways having less directly studied relevance to ALS (for example, neurodevelopmental processes and pathways such as gastrulation, neural tube development, and Shh pathways), may be of interest for further investigation.

It may be of interest to note that certain processes, despite their heavy implication in ALS, were not more prominent among gene set associations. For example, mechanisms of DNA damage and repair, although implicated in several common and less frequent ALS mutations [92], were only indirectly represented in the gene set associations: the CDMAC pathway, reported above in the immune response findings, relates to cadmium genotoxicity. Processes relating to RNA/protein transport, ER stress, and autophagy, were also not prominent, although several gene sets related to these were present within other functional categories: for example, vesicle-mediated transport to the plasma membrane, the anterograde dendritic transport of neurotransmitter receptor complex and the PPAR-alpha pathway, all have association to ALS (each being listed among one or more of the functional categories reported in Table 3, Table 4, Table 5, Table 6 and Table 7), as well as an enrichment to early and late endosomes in the shared genes of the interacting terms KEGG Gap junction and BioCarta GPCR pathways.

ALS is a neurodegenerative disease characterized primarily by the loss of the upper and lower motor neurons. However, the mechanisms that affect motor neurodegeneration have not been fully elucidated. A pathological hallmark of ALS is the aberrant misfolding, aggregation, and deposition of protein inclusions formed by TAR DNA-binding protein of 43 kDa (TDP-43), Cu/Zn superoxide dismutase (SOD1), or fused in sarcoma (FUS) in motor neurons [76]. The significant gene sets show several connections to motor neuron pathology and the major ALS disease-associated protein of TAR DNA-binding protein of 43 kDa (TDP-43). The nervous system category included five gene sets that reached robust statistical significance (FDR < 0.05), including the BioCarta CREB, Shh, DREAM, CK1 and AGPCR pathways. The BioCarta CREB (cyclic AMP-responsive element binding) gene set is highly associated with ALS (FDR = 0.039) and highly popular in the ALS network presented here, and has been recently linked to altered expression of *TDP-43* [93]. A recent study suggested that *TDP-43* RNA targets are enriched in signaling pathways of the CREB transcription factor, and that TDP-43 dysfunction inhibits the activation of CREB, restricting dendritic complexity [93]. Altered *TDP-43* expression results in reduced dendritic growth [94] and in vivo experiments inducing altered dendritic growth result in disrupted neuronal connectivity and cell communication, which may be causal of ALS [95,96]. A progressive inhibition of CREB activation has been observed in human *C9ORF72*-mutant motor neurons while also exhibiting an underexpression of synaptic genes and synaptic loss [97]. The DREAM (downstream regulatory element antagonistic modulator) (FDR = 0.040) transcriptional regulator is expressed in spinal cord neurons and has been linked to ALS-associated neuronal death and has been found to be up-regulated in *SOD1* mice and ALS patients [98]. DREAM has been associated with increased apoptotic activity towards motor neurons and astrocytes induced by a progressive calcium-dependent excitotoxicity that ultimately leads to neuronal damage and motor neuron loss in ALS [98]. In addition, the highly associated Casein kinase 1 (CK1) gene set in our analysis, a dopaminergic signaling pathway in the neostriatum, has been reported to have links with motor neuron degenerative diseases including ALS [72]. Specifically, there is evidence of abnormal TDP-43 hyperphosphorylation in the brain of ALS patients and it has been shown that CK1 mediates this hyperphosphorylation on TDP-43, highlighting an important role of CK1 in ALS and other neurodegenerative diseases  [72,99].

Prominent patterns of ALS-associated gene sets were observed within the immune response category, related mainly to inflammation, T cell activation/regulation, T cell receptor signaling processes, cytokine activities and innate immunity. Despite that ALS has not been generally considered a disease affected primarily by autoimmunity and/or immunodeficiency, there is increasing evidence that immune dysregulation and neuroinflammation affect its onset and progression [100,101]. Neuroinflammation is a process that is generally observed in the context of infection, injury or degeneration, and it is described by the reaction of glial (astrocytes, microglia) and infiltrating immune cells (monocytes, neutrophils, lymphocytes) with cells of the central nervous system [102]. Previous studies have highlighted neuroprotective and neurotoxic phenomena that both derive from inflammation and appear to be specific to the progression phase of ALS [101]. We identified key immune response regulation pathways that have neuroprotective and immunomodulatory roles that prevent inappropriate and hazardous T-cell activation that could lead to autoimmune pathogenesis. T cells play a role in ALS pathology and have been identified in autopsy tissues from ALS patients [102,103]. Such gene sets include the most strongly ALS-associated gene set in our analysis, the BioCarta CSK pathway (FDR = 0.009), which is implicated in the inhibition of T-cell receptor signaling and T-cell activation, BioCarta VIP (vasoactive intestinal peptide) (FDR = 0.049) and CTLA-4 (cytotoxic T-lymphocyte antigen-4) (FDR = 0.054). Interestingly, the VIP pathway inhibits the apoptosis of activated T cells through two neuropeptides VIP and PACAP (pituitary adenylate cyclase-activating polypeptide); these two neuropeptides are key regulators of the function of microglial cells during myelin degeneration and have been associated with a number of neurodegenerative diseases [34]. Microglia and astrocytes have been well studied for their role in neurodegeneration in ALS and their inflammatory and potentially neuroprotective attributes after activation, which consist of one of the hallmarks of ALS pathology  [100,104]. The ALS-associated BioCarta NFAT pathway (FDR = 0.069) is also associated with the activation of T cells, and the NFAT transcription factor has been found to be involved with the regulation of pro-inflammatory responses in cultured murine microglia [74].

It has been previously proposed that an innate immune response (rather than an adaptive immune response) is responsible for ALS-specific neuroinflammation [102,103]. Innate immune cells including monocytes, neutrophils, dendritic cells, macrophages and mast cells have been linked to ALS pathology, while lymphocytes and monocytes have been linked to immune dysregulation [100,102]. Our GS-GWAS analysis identified several ALS-associated pathways related to the innate immune system and to circulating immune cells, including the BioCarta monocyte (FDR = 0.040), lymphocyte (FDR = 0.046), NK (natural killing) cells (FDR = 0.019) and neutrophil (FDR = 0.226) pathways. In addition, the CFTR pathway (FDR = 0.046) was also found to be significantly enriched, associated among others with the innate immune system and expressed in macrophages and neutrophils [37,38]. This may be relevant to previous suggestions that immune cell infiltration is increased in ALS patients [100]. Several significant gene sets in the analysis were also related to inflammatory cytokines. Cytokine activities have been previously associated with ALS neuroinflammation pathology and to a possibly autoinflammatory state [100,101,103].

Another interesting observation was a strong ALS association to oxidized phospholipid pathways, which have been recently proposed as novel mediators of neurodegeneration [105]. Oxidized phospholipids have been linked to pro-inflammatory and anti-inflammatory responses through the stimulation of endothelial cells producing inflammatory cytokines as well as in acute and chronic microbial infections and oxidative stress [42,43]. Inflammation and oxidative stress can lead to a reactive oxygen species (ROS)-induced lipid peroxidation, generating oxidized phospholipids [105,106]. Oxidized phosphatidylcholines (OxPCs) have been proposed as novel neurotoxins requiring neutralization by microglia, and biomarkers of oxidative stress [105]. In addition, OxPCs have been detected in lesions of ALS patients, among other neurodegenerative disorders, including multiple sclerosis, frontotemporal lobe dementia and spinal cord injury [105]. The fourth most strongly ALS-associated gene set of our analysis (and second in the immune-response category) was the Gargalovic response to oxidized phospholipids black up (FDR = 0.032). This gene set refers to a network module containing genes that are up-regulated in primary aortic endothelium cells after exposure to oxidized phospholipids [42]. The authors who generated this curated gene set ((black module) found that it was functionally enriched in genes involved in Prion disease [42]. This is apparent also from our enrichment map, as the only connection in the ALS network (as shown in Figure 5) of the Gargalovic response to oxidized phospholipids black up gene set is to the BioCarta Prion pathway (FDR = 0.054). The Prion pathway has been associated with neurotoxicity and neurodegeneration. Prion diseases are a group of fatal neurodegenerative diseases, caused by the misfolding and self-propagation of prion proteins and it has been proposed that ALS shares parallel prion-like mechanisms involved in the disease pathogenesis [75,76]. The third most highly significant gene set of the GS-GWAS analysis, PID RhoA (Ras homolog gene family member A) pathway (FDR = 0.022), which is primarily linked to the cytoskeleton and to cell-signaling processes, has also been associated with prion-related neurodegeneration [68].

We also report a noteworthy ALS association to the peroxisome proliferator-activated receptor alpha (PPAR-alpha) pathway, which displays multiple interesting patterns in the current analysis. PPARs belong to the family of ligand-regulated nuclear receptors and can be activated, among other lipid categories, by oxidized phospholipids. The lipid metabolism gene set BioCarta PPAR-alpha pathway (FDR = 0.046) was among the top ALS-associated results and has the second highest degree within the ALS enrichment network. Specifically, the PPAR-alpha phosphoprotein has been linked to immune and inflammation responses, autophagy in human microglia, oxidative phosphorylation, regulation of the oxidative metabolism of skeletal muscle and energy homeostasis [61,85,87]. PPAR-alpha has been proposed as a putative novel therapeutic target in various diseases, including ALS, slowing the progression of the disease [107,108]. Specifically, an in vivo ALS study demonstrated that the activation of PPAR-alpha results in neuroprotection, neuroinflammation reduction, and neurodegeneration blocking [108]. *PRKCB* was the highest ALS-associated gene within the BioCarta PPAR-alpha pathway (FDR = 0.037). PRKCB is a member of the serine- and threonine-specific protein kinases family, and it has been associated with various processes, including, among others, immune response homeostasis and initiation [109], lipid peroxidation and induced ferroptosis [110] and Alzheimer’s disease pathogenesis [111].

The BioCarta PPAR-alpha pathway is connected with the equally highly significant BioCarta cystic fibrosis transmembrane conductance regulator (CFTR) pathway (FDR = 0.046), sharing an almost complete overlap of first significant neighbors within the lipid subnetwork. Putative links between ALS and cystic fibrosis (CF) have been noted previously, with TAR DNA-binding protein 43 (TDP-43) dysfunction being linked to the underlying pathology of both diseases [112].

Lastly, our results show several neurodevelopmental pathways that may have a role in ALS pathology. In fact, the most strongly significant ALS-associated gene set was the BioCarta mPR (membrane progesterone receptor) pathway (FDR = 0.009) which involves oocyte maturation by progesterone. MPRs have been previously associated with neuroprotective functions in neurons [51]. Progesterone is a pleiotropic regulator of neurons and glial cells and has been linked with decreased neuroinflammation, neuroprotection, neuronal survival and antioxidant effects in ALS mouse models [113]. In addition, the BioCarta Sonic Hedgehog (Shh) pathway (FDR = 0.040) is involved in several important neuron developmental processes, including, among others, the development of the neural tube, motor neurons, the regulation of CNS polarity, neuronal regeneration and proliferation as well as exhibiting a cytoprotective role against oxidative and excitotoxic stress [52,53,54]. There is evidence of inhibition of the Shh signaling pathway in ALS cerebrospinal fluid samples [114], as well as cytoprotective effects against oxidative stress in in vitro models of ALS, suggesting a potential role of the Shh pathway in ALS pathology [115].

### 3.3. Gene Set Interaction Analysis

The interaction analysis showed that the BioCarta PPAR-alpha pathway interacts with CREB (cyclic AMP response element-binding protein 1) (*p*-value = 0.014) and GPCR (G-protein coupled receptor) pathways (*p*-value = 0.019). The CREB pathway is the most highly enriched gene set within the nervous system category (FDR = 0.039) and the most popular node within the ALS network. CREB affects a variety of signaling cascades including the ERK1/2, MAPK, PI3K/AKT, CaMK, PKC, and PKA pathways [59]. The shared genes within both interactions as well as the shared genes between the BioCarta PPAR-alpha and the CFTR pathways are protein-coding genes that are members of the serine/threonine protein kinase family, coding various subunits of cyclic AMP (cAMP) dependent protein kinase. This set of genes has been found to be significantly enriched among others to the gene ontology biological processes, “activation of protein kinase A activity” and “negative regulation of cAMP-dependent protein kinase activity”. The cAMP-dependent protein kinase A (PKA) pathway has been associated with the rescued mislocalization of TDP-43—one of the main pathological hallmarks for neurodegeneration in ALS [116]. The same study proposed PKA as a novel drug target for ALS [116]. In addition, an earlier study showed a statistically significant elevated expression of the protein kinase A in fractions of spinal cord tissue of ALS patients [117].

### 3.4. Limitations

Several limitations should be taken into account in interpreting the results of the current work. Despite the fact that we employed the largest ALS individual-level genomic data with European descent releases to date, the power of GWAS is highly dependent on sample size, so the genotyping of larger ALS cohorts could potentially unravel further genetic associations and consequently in-depth knowledge about the pathology of the disease. We did not consider very rare variants (MAF < 0.5%), as such variants are typically removed from GWAS analyses, being considered putative false positives. Rare variants have been proposed to play a key role in ALS. As with any GWAS study, it is possible to identify spurious associations, and much of our SNP-level methodology, including extensive quality control filtering, and careful consideration of population structure, was devoted to the minimization of this risk. In addition, the MAGMA tool has features taking into account gene density (representing the relative level of linkage disequilibrium between SNPs in each gene), gene size (number of SNPs), and gene–gene correlations, but these do not eliminate the possibility of identifying spurious associations. Finally, the gene-set analysis results are highly dependent on the current knowledge of biological pathways stored in gene-set annotation databases. Our gene-level analysis considers only protein-coding genes and their neighboring regulatory regions by including upstream and downstream windows of 20 kb. In addition, our analysis does not include multi-locus events in the genome. The incorporation of non-coding loci, regulatory elements, as well as epistatic events in the genome, has been proposed as a way to gain insight into disease mechanisms  [16].

## 4. Materials and Methods

### 4.1. Datasets

To form the ALS-control cohort, two restricted access dbGaP projects have been used. The first project refers to the largest release of European descent of ALS individual-level genotype data to date, with accession number phs000101.v5.p1 and contains genotype data of multiple genomic platforms for a total of 15,480 samples including patients suffering from amyotrophic lateral sclerosis diagnosed using the El Escorial criteria and healthy controls [9,26]. From this project, we collected individual-level genotype data of 471,303 SNPs from 12,319 people (7015 males, 5214 females, 90 ambiguous) of which 10,047 were cases, 2181 were controls and 91 phenotypes were missing. These data were further processed and filtered using quality control strategies described in subsequent sub-chapters. To increase the power of our analysis, we collected a second control cohort from dbGaP with accession number phs000428.v2.p2 (Health and Retirement Study). In order to keep a homogeneous cohort, we excluded from the latter study all the self-reported African-American samples, leaving 13,210 samples containing 2,315,518 variants for subsequent analysis. The age of the recruited individuals from the Health and Retirement Study ranged from 55 to 79 years of age.

### 4.2. Genomic Quality Control Analysis

We used snpQT (v0.1.7) as the main software for quality control (QC), population stratification, association analysis, pre-imputation and post-imputation QC. snpQT is an automatic Nextflow software that ensures reproducibility and scalability, with an easy-to-use design [118].

To avoid potential batch effects, we applied sample and variant QC to each cohort, separately, using the snpQT --qc workflow. Sample QC workflow in both cohorts included first the removal of variants with a very poor quality (call rate < 90%)—which would be removed in subsequent analysis anyway to avoid removing extra samples—and then the removal of samples with sex discrepancies, a missing phenotype (i.e., case/control), poor quality (call rate < 98%), and extreme heterozygosity (deviating 3 standard deviation units from the mean) as well as duplicated and cryptic relatedness samples (relatedness > 12.5%). When sample QC was completed, the next step was variant QC workflow, which included the removal of variants with poor quality (call rate < 98%), deviation from the Hardy–Weinberg equilibrium (*p*-value < 10${}^{-7}$), with a minor allele frequency (MAF) less than 1% and with a significant difference in call rate between cases and controls (*p*-value < 10${}^{-7}$). The latter check was not carried out for the aging cohort, as all the samples are controls.

When sample and variant QC were completed, the next step was to merge the two cohorts keeping only common variants between the two datasets. Before merging the datasets, we assured that they were aligned in the same human genome build, flipped variants to the forward strand where necessary, and removed any palindromic SNPs, using PLINK [119] and snpflip (https://github.com/biocore-ntnu/snpflip accessed on 15 January 2023, version 0.0.6). After the ALS and aging cohorts were merged, we used snpQT (v0.1.7) [118] to perform sample QC, population stratification to remove sample outliers, and variant QC, combining --qc and --pop_strat workflows. The checks and the applied thresholds on the merged cohort included the removal of samples with the following variants: very poor quality (call rate < 90%); poor quality (call rate < 98%); extreme heterozygosity (deviating 3 standard deviation units from the mean); duplicated samples; cryptic relatedness (PLINK king-ship coefficient > 0.125); outlier samples using EIGENSOFT (sigma threshold = 4) and variants with poor quality (call rate < 98%); variants that deviate from the Hardy–Weinberg equilibrium (*p*-value < 10${}^{-7}$); rare variants with a minor allele frequency (MAF) less than 1% and with a significant difference in call rate between cases and controls (*p*-value < 10${}^{-7}$).

While a thorough quality control procedure was carried out for each dataset separately, it was important to address errors and batch effects that usually emerge when merging multiple datasets deriving from different analytical platforms genotyped at different times and places [120]. To further handle potential batch effects between the two cohorts, we followed two main strategies. First, we labeled cases as “missing”, controls from the ALS cohort as “cases” and controls from the aging cohort as “controls”, performed logistic regression to this artificially labeled cohort and removed 3519 variants with high statistical association (*p*-value < 10${}^{-6}$). Lastly, we removed 62,309 variants with a statistically significant differential case-control call rate (*p*-value < 10${}^{-6}$) on a second artificial merged dataset, labeling all the samples from the ALS cohort and the aging cohort as “cases” and “controls”, respectively.

To maximize the size of our final cohort through meta-analysis, we collected the excluded outlier samples after population stratification and followed a separate cycle of quality control analyses, and we refer to these as the minor case/control cohort. This minor case/control cohort consists of 399,837 variants and 3058 samples of which 672 and 2386 samples are cases and controls, respectively.

For the minor case/control cohort, we removed any samples which overlapped with non-European ancestry cohorts. Then, we followed the same sample/variant QC, population stratification and batch effects correction strategies as we did for our major case/control cohort, described above.

### 4.3. Imputation

To increase the number of SNPs in the two processed ALS-control datasets, we used the Sanger imputation service provided by the Wellcome Sanger Institute [121]. Before uploading the ALS-control cohorts to the Sanger imputation server, we employed the snpQT pre-imputation quality control workflow [118]. The snpQT pre-imputation quality control aligns with the Sanger imputation service prerequisites, including checks for the chromosome codes, removing duplicated variants, and lastly, using bcftools’s plug-in +fixref to correct for remaining discrepancies (e.g., fixing the reference allele with respect to a FASTA 1000 genome reference dataset).

In the Sanger imputation service, we chose the Haplotype Reference Consortium [121] as a reference panel, EAGLE2 [122] as a phasing software and positional Burrows–Wheeler transform (PBWT) [123] as an imputation algorithm. We further applied post-imputation quality control on the imputed dataset, using the snpQT post-imputation quality control workflow. This workflow included the removal of rare variants with a minor allele frequency (MAF) less than 0.5%; variants with an INFO score less than 0.4; duplicated variants in terms of their ID; and variants that corresponded to the same SNP (duplicated records, merged variant ids among multiple dbGaP versions).

### 4.4. Genome-Wide Association Analysis

We used the snpQT GWAS workflow for both imputed cohorts to acquire SNP associations to the ALS phenotype [118]. For this purpose, snpQT uses PLINK2’s generalized linear regression model and offers the option of covariates. We used gender and the first three principal components to adjust for fine-scale population structure as covariates for all cohorts.

**Figure 9:**
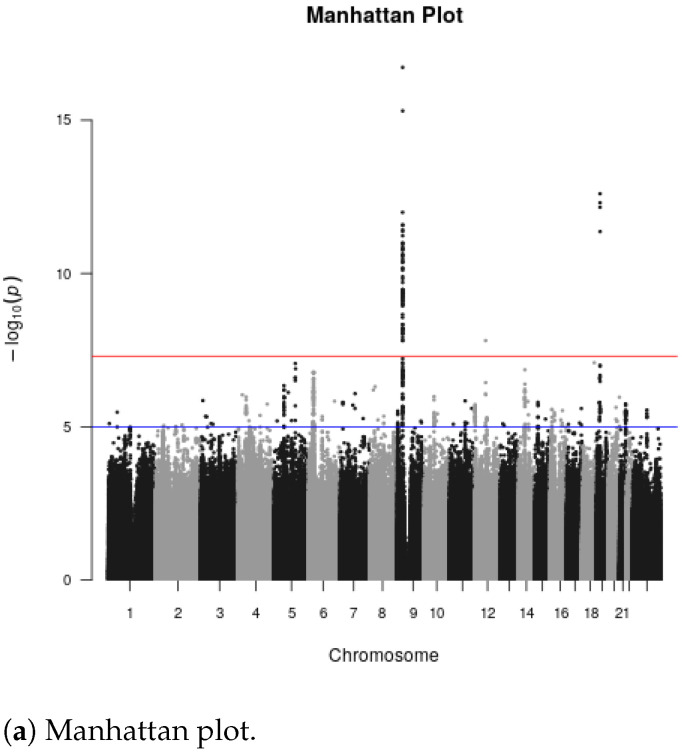

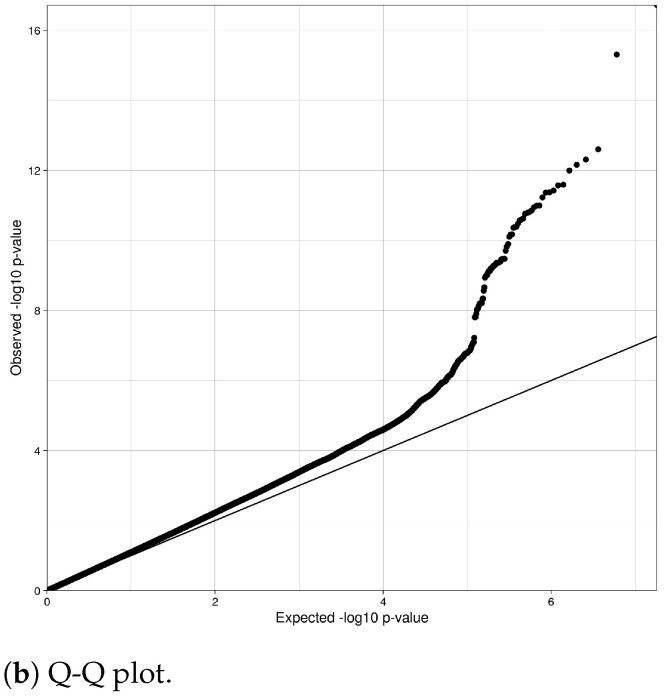
Manhattan plot (**a**) and Q-Q plot with a lambda coefficient equal to 1.12 (**b**) of the ALS-control major cohort, including 20,981 samples and 9,056,962 variants. Manhattan plot shows the association *p*-values of each genetic variant (y-axis) and their corresponding genomic positions (x-axis). Red and blue lines indicate −log(10${}^{-8}$) and −log(10${}^{-5}$) thresholds of genome-wide significant association.

**Figure 10:**
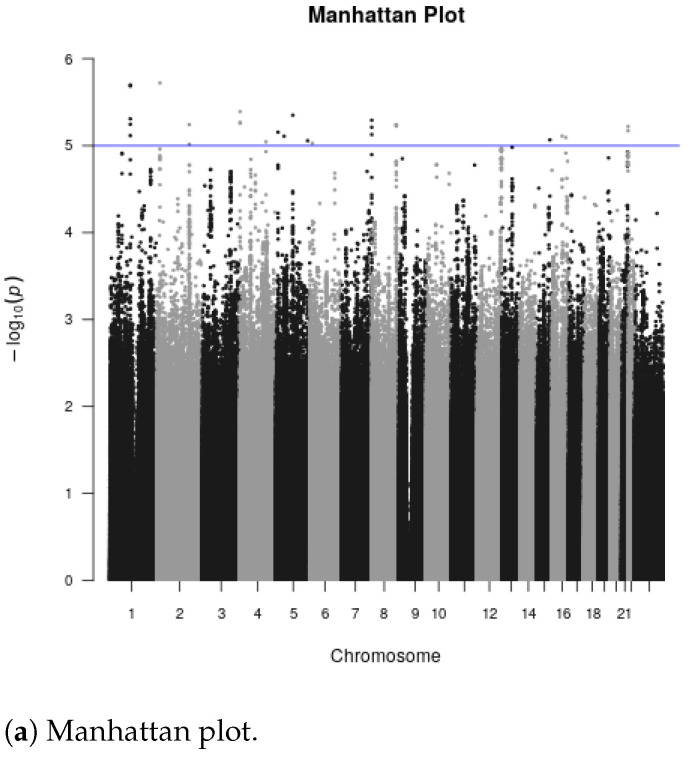

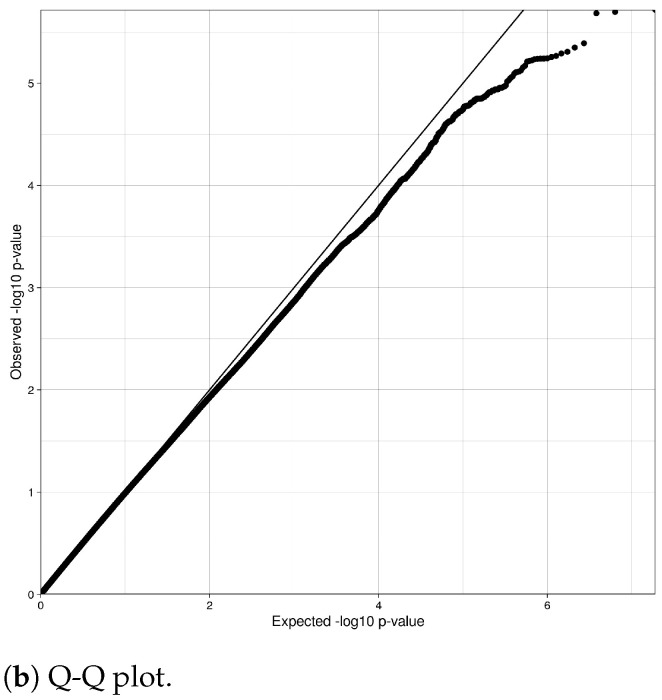
Manhattan plot (**a**) and Q-Q plot with a lambda coefficient equal to 1.01 (**b**) of the ALS-control minor cohort, including 1058 samples and 9,492,483 variants. Manhattan plot shows the association *p*-values of each genetic variant (y-axis) and their corresponding genomic positions (x-axis). Blue line indicates −log(10${}^{-5}$) genome-wide significant association.

Following post-imputation quality control, the major case/control cohort included 9,056,962 variants genotyped from 20,981 samples of which 8802 were cases and 12,179 controls. In terms of gender, the major case/control cohort included 10,510 females and 10,471 males. The minor case/control cohort consisted of 9,492,483 variants and 1058 samples: 442 cases and 616 controls; 475 females and 583 males. In Figure 9 and Figure 10, the Q-Q (Quantile-Quantile) and Manhattan plots for the two imputed ALS-control cohorts, respectively, are shown, generated from snpQT. The Manhattan plot illustrates the association *p*-values (shown on the y-axis), and the chromosomal positions of the tested genetic variants (shown on the x-axis), whereas the Q-Q plot shows the relationship between the observed and the expected quantile *p*-values of the ALS/control cohort under a normal distribution. Moreover, the Q-Q plot shows the lambda genomic inflation coefficient (calculated as median 1df chi-square stat/0.456), used to control false positive results and thus, reduce prediction errors.

### 4.5. Annotation and Gene Analysis

To discover ALS-associated genes and gene sets, we used the command-line software multi-marker analysis of genomic annotation (MAGMA version 1.10) [22].

Prior to the gene analysis, we performed an annotation step for all cohorts, mapping SNPs into genes based on their genomic location, using the NCBI human genome build 37 location file available at MAGMA website and the resulting .bim PLINK files of the ALS-control cohorts. We chose a window of 20 kb upstream and downstream of each gene in order to also include regulatory elements in our analysis.

The gene-level annotation step, mapping each genetic variant to a gene, yielded 4,788,553 and 5,023,961 variants for the major and minor ALS-control cohorts, which were mapped to at least one gene, respectively. MAGMA does not support analyses including the Y chromosome; therefore, no SNPs were mapped to chromosome Y.

The next step was gene analysis, where multiple SNP *p*-values are summarized into genes using the MAGMA SNP-wise multi-model. The multi SNP-wise model combines two different genetic architectures of SNP-wise mean and SNP-wise top,1 models, generating one aggregate multi *p*-value. The SNP-wise mean model is more sensitive to the mean association of the mapped SNPs of a gene, whereas SNP-wise top,1 considers only the highest associated SNP mapped to each gene. Additional to the calculation of the gene *p*-values, the gene correlations are estimated while accounting for linkage disequilibrium (LD) phenomena [22,124]. Gene analysis was performed on the two GWAS datasets, providing the total number of samples, as well as the number of female and male samples, for each GWAS cohort.

### 4.6. Gene Meta-Analysis

To utilize the power of both of the collected and analyzed ALS-control cohorts, we performed a meta-analysis at the gene level to combine the gene level results of the two cohorts (major and minor case/control cohorts) and then gene-set analysis, using the command-line software multi-marker analysis of genomic annotation (MAGMA version 1.10) [22]. MAGMA uses the weighted Stouffer’s Z method in order to combine the Z-scores for each gene across cohorts. MAGMA does not require a perfect overlap of genes between cohorts. The software checks that each gene across the cohorts has the same genomic locations, and then the meta-analysis for each gene is performed using any cohort available for the particular gene [22]. The meta-analysis yielded a total of 19,242 genes, from 22,039 samples of 9244 ALS cases (3852 females and 5392 males) and 12,795 healthy controls (7133 females and 5662 males).

### 4.7. Gene-Set Analysis

For gene-set analysis, the MAGMA competitive model was employed, testing if there is a statistically significant combined association of the genes within a gene set with the phenotype of interest, in comparison with the genes not in the gene set [19,22]. To extract groups of gene sets, we used the Molecular Signatures Database (MSigDB v7.5) [24,25]. In Table 10, the categories of the 31,454 collected gene sets are summarized.

### 4.8. Interaction Analysis

Interaction gene-set analysis was performed on all possible pairs of the 145 gene sets that were statistically significant in the previous analysis, and the subset of common genes that they share. By performing interaction gene-set analysis for each pair of these gene sets using MAGMA [22], we aimed to determine whether each of these gene sets had a statistically significant association with the ALS trait in its own right, or whether the significance of one or both of each pair may be derived from the genes shared by the two.

In order for MAGMA to carry out interaction analysis between a pair of gene sets, there must be an overlap between the sets without one set being completely contained within the other. By default, MAGMA will consider an interaction term between two gene sets as valid (i.e., testable) if it contains at least 25 genes, with each gene set containing at least 25 unique genes not within the interaction term, and with these unique genes representing at least 10% of its gene set. Given that this analysis is exploratory, these parameters were set wider than the default in order to enable MAGMA to identify potential significant interactions, even for relatively small overlaps between gene sets. The parameters were set so that the interaction terms contained at least 10 genes, with each interacting gene set containing at least 10 unique genes which are not present within the interaction term, and that represented at least 1% of the original gene set.

### 4.9. Enrichment Networks

For the visualization of the gene-set analysis results, we used Cytoscape (version 3.9.0) to create enrichment maps [125]. We applied a cut-off of 0.25 false discovery rate (FDR) to retain the highest statistically significant gene sets. An enrichment map is a network where each node represents an ALS-associated gene set. Each node has a specific size depicting the number of genes that are included in each gene set and that have been used for the gene-level analysis in MAGMA. Furthermore, we colored the nodes with their corresponding FDR as well as a meta-annotation category based on major biological processes: apoptosis and cell survival, homeostasis/cell metabolism, immune response, cell cycle, regulation of transcription/translation, muscle, cytoskeleton, lipid metabolism, nervous system, and developmental pathways. The biological categorization of the gene sets was carried out after curation of related literature and biological information resources such as UniProt [126], GeneCards [127], the molecular signatures database [24,25], AmiGO 2 [128,129,130] and KEGG [131]. Two nodes are connected through an edge that represents a minimum 10% overlap (similarity) between the two gene sets. The similarity is represented by the average of the Jaccard coefficient and the overlap coefficient. The formulas of the two similarity coefficients for gene sets A and B are the following:

- Jaccard coefficient = [size of (A intersect B)]/[size of (A union B)].
- Overlap coefficient = [size of (A intersect B)]/[size of (minimum(A, B))].

Each edge is characterized by a certain width, based on the overlap of the connected gene sets/ nodes, expressed by the combined similarity coefficient.

Lastly, we performed a network topology analysis using the Cytoscape (v3.9.0) analyze network tool, through which we obtained information about the number of nodes and edges within each network, and the nodes’ centrality, expressed by the degree of each node. The degree of each node represents the number of undirected edges that it contains and reflects its popularity within the network.

## 5. Conclusions

This study identified significant gene set associations to ALS that are related to a variety of biological categories, including, immune response, neuron and embryonic development, lipid metabolism, nervous and muscle system processes, summarized in Figure 8.

Prominent patterns of gene set significance relating to autoimmunity, immune dysregulation, and neuroinflammation were observed. We report the neuroprotective and immunomodulatory activities of T-cell receptor signaling and T-cell activation, inflammatory cytokines, innate immune responses and immune cell infiltration processes as associated with ALS. These results are in accordance with previous studies that provided supporting evidence for ALS-specific neuroinflammation and immune dysregulation mechanisms [102,103]. The potential causal implications of the gene set association may have relevance to the ongoing discussion of the roles of immune dysregulation and neuroinflammation in ALS, and whether they contribute actively to the progression of the disease, or if they are a consequence of motor neuron degeneration and injury [100,101].

Oxidative stress and protein degradation are established ALS-associated processes. The GS-GWAS results provide potential links of these to motor neurodegeneration and to the ALS disease-associated protein, TAR DNA-binding protein of 43 kDa (TDP-43), mainly deriving from nervous system-related gene sets, such as the BioCarta CREB, Shh, DREAM, CK1 and AGPCR pathways. Oxidized phospholipid pathways, generated by a ROS-induced lipid peroxidation and prion-related mechanisms, are ALS-associated, and may also play a role in the neurotoxicity and neurodegeneration of the ALS pathology.

Competitive and interaction gene-set analyses revealed an intriguing pattern of functional relationships of the proliferator-activated receptor alpha (PPAR-alpha) pathway. PPAR-alpha has been linked among others to immune and inflammation responses, autophagy in human microglia, regulation of the oxidative metabolism of skeletal muscle, as well as key roles in neuroprotection, neuroinflammation reduction, and blocking of neurodegeneration [61,85,87,108]. The interaction analysis revealed an interaction term of the PPAR-alpha gene set with the CREB and GPCR pathways. PPAR-alpha, CREB and GPCR pathways were among the top associated gene sets in our analysis, with CREB and PPAR-alpha having the highest number of neighbors in the ALS network. The shared genes of the three gene sets were enriched with protein kinase A (PKA) and cAMP-dependent protein kinase activities. The cAMP PKA pathway has been associated with rescued mislocalization of TDP-43—one of the main pathological hallmarks for neurodegeneration in ALS [116].

Lastly, we note several neurodevelopmental pathways that our analysis suggests could have potential causal roles in ALS pathology, and have previously been linked to the disease through molecular studies. The top associated gene set, the mPR (membrane progesterone receptor) pathway (FDR = 0.009), has been associated with neuroprotective functions in neurons [51], decreased neuroinflammation, neuroprotection, neuronal survival and antioxidant effects in ALS mouse models [113]. The Sonic Hedgehog (Shh) pathway (FDR = 0.040) was present within the generated ALS gene set networks, and has been suggested to have a cytoprotective role against oxidative and excitotoxic stress in ALS pathology [52,53,54,115].

A future direction of this work could be to combine expression quantitative trait locus (eQTL) data and genomic data, using summary data-based Mendelian randomization (SMR), to test the chance that genetic variants that increase risk of a disease do so through modifying gene expression [132]. Finally, employing machine learning models to predict multi-locus interactions that are associated with ALS could contribute toward understanding the underlying mechanisms of this devastating disease [16].

## Figures and Tables

**Table 1:** The 6 highly significant genes that are associated with ALS in the ALS-control cohort including 22,039 samples, sorted by their FDR (FDR < 0.01). Each highly significant gene is described by its gene symbol, chromosome, number of mapped SNPs, the MAGMA multi-model *p*-value and FDR. We further note that the 4 top genes also pass the Bonferroni multiple testing correction (alpha = 0.05; *p*-value < $2.58\times 10^{-6}$). FDR: false discovery rate, SNP: single nucleotide polymorphism.

Gene	Chromosome	No. SNPs	*p*-Value	FDR
*MOB3B*	9	979	9.20 $\times \;10^{-17}$	1.77 $\times \;10^{-12}$
*IFNK*	9	206	4.14 $\times \;10^{-14}$	3.98 $\times \;10^{-10}$
*C9ORF72*	9	289	3.90 $\times \;10^{-12}$	2.50 $\times \;10^{-8}$
*UNC13A*	19	487	2.37 $\times \;10^{-11}$	1.14 $\times \;10^{-7}$
*ADARB1*	21	726	3.13 $\times \;10^{-7}$	1.20 $\times \;10^{-3}$
*KIF5A*	12	142	2.44 $\times \;10^{-6}$	0.78 $\times \;10^{-2}$

**Table 2:** The 24 significant gene sets that are associated with ALS, sorted by their FDR (FDR cut-off < 0.05). Each significant gene set is described by its FDR, degree i.e., number of undirected edges within the ALS enrichment network containing gene sets with FDR < 0.25, and their corresponding number of genes. The vast majority of the significant gene sets are highly popular. FDR: false discovery rate.

Gene sets	*p*-Value	FDR	Degree	No. Genes
BIOCARTA_MPR_PATHWAY	6.27 $\times \;10^{-5}$	9.15 $\times \;10^{-3}$	38	21
BIOCARTA_CSK_PATHWAY	5.02 $\times \;10^{-5}$	9.15 $\times \;10^{-3}$	29	22
PID_RHOA_PATHWAY	1.16 $\times \;10^{-4}$	2.28 $\times \;10^{-2}$	11	45
GARGALOVIC_RESPONSE_TO_				
OXIDIZED_PHOSPHOLIPIDS_BLACK_UP	9.54 $\times \;10^{-6}$	3.22 $\times \;10^{-2}$	1	35
GOMF_CYCLIC_NUCLEOTIDE_				
DEPENDENT_PROTEIN_KINASE_ACTIVITY	6.70 $\times \;10^{-5}$	3.88 $\times \;10^{-2}$	26	10
GOMF_CYCLIC_NUCLEOTIDE_BINDING	6.59 $\times \;10^{-5}$	3.88 $\times \;10^{-2}$	21	38
GOMF_DIOXYGENASE_ACTIVITY	6.11 $\times \;10^{-5}$	3.88 $\times \;10^{-2}$	5	94
BIOCARTA_CREB_PATHWAY	4.05 $\times \;10^{-4}$	3.94 $\times \;10^{-2}$	50	22
MIR12119	1.68 $\times \;10^{-5}$	4.00 $\times \;10^{-2}$	1	185
BIOCARTA_IGF1R_PATHWAY	9.71 $\times \;10^{-4}$	4.03 $\times \;10^{-2}$	45	23
BIOCARTA_BAD_PATHWAY	8.07 $\times \;10^{-4}$	4.03 $\times \;10^{-2}$	37	25
BIOCARTA_DREAM_PATHWAY	1.10 $\times \;10^{-3}$	4.03 $\times \;10^{-2}$	34	13
BIOCARTA_SHH_PATHWAY	6.60 $\times \;10^{-4}$	4.03 $\times \;10^{-2}$	28	16
BIOCARTA_MONOCYTE_PATHWAY	9.34 $\times \;10^{-4}$	4.03 $\times \;10^{-2}$	11	11
MIR4286	3.76 $\times \;10^{-5}$	4.47 $\times \;10^{-2}$	0	92
BIOCARTA_PPARA_PATHWAY	1.71 $\times \;10^{-3}$	4.63 $\times \;10^{-2}$	42	52
BIOCARTA_CK1_PATHWAY	1.71 $\times \;10^{-3}$	4.63 $\times \;10^{-2}$	26	16
BIOCARTA_CFTR_PATHWAY	1.81 $\times \;10^{-3}$	4.63 $\times \;10^{-2}$	25	11
BIOCARTA_LYMPHOCYTE_PATHWAY	1.91 $\times \;10^{-3}$	4.63 $\times \;10^{-2}$	11	9
BIOCARTA_TCR_PATHWAY	2.49 $\times \;10^{-3}$	4.85 $\times \;10^{-2}$	39	44
BIOCARTA_AGPCR_PATHWAY	2.56 $\times \;10^{-3}$	4.85 $\times \;10^{-2}$	36	11
BIOCARTA_STATHMIN_PATHWAY	2.30 $\times \;10^{-3}$	4.85 $\times \;10^{-2}$	31	20
BIOCARTA_GHRELIN_PATHWAY	2.66 $\times \;10^{-3}$	4.85 $\times \;10^{-2}$	1	13
BIOCARTA_VIP_PATHWAY	2.87 $\times \;10^{-3}$	4.93 $\times \;10^{-2}$	33	26

**Table 3:** The 26 significant gene sets that are related to immune response, sorted by their FDR (FDR cut-off < 0.24 and *p*-value < 0.043). Each significant immune-response gene set is described by its *p*-value, FDR, degree, i.e., number of edges within the immune-response ALS enrichment network containing gene sets with FDR < 0.25, and their corresponding number of genes. We note that the vast majority of significant gene sets are highly popular. FDR: false discovery rate.

Gene Sets	*p*-Value	FDR	Degree	No. Genes
BIOCARTA_CSK_PATHWAY	5.02 $\times \;10^{-5}$	9.15 $\times \;10^{-3}$	20	22
GARGALOVIC_RESPONSE_TO				
_OXIDIZED_PHOSPHOLIPIDS_BLACK_UP	9.54 $\times \;10^{-6}$	3.22 $\times \;10^{-2}$	0	35
BIOCARTA_MONOCYTE_PATHWAY	9.34 $\times \;10^{-4}$	4.03 $\times \;10^{-2}$	4	11
BIOCARTA_CFTR_PATHWAY	1.81 $\times \;10^{-3}$	4.63 $\times \;10^{-2}$	16	11
BIOCARTA_LYMPHOCYTE_PATHWAY	1.90 $\times \;10^{-3}$	4.63 $\times \;10^{-2}$	4	9
BIOCARTA_TCR_PATHWAY	2.49 $\times \;10^{-3}$	4.85 $\times \;10^{-2}$	16	44
BIOCARTA_VIP_PATHWAY	2.87 $\times \;10^{-3}$	4.93 $\times \;10^{-2}$	20	26
BIOCARTA_CTLA4_PATHWAY	3.53 $\times \;10^{-3}$	5.42 $\times \;10^{-2}$	7	22
BIOCARTA_TCRA_PATHWAY	4.17 $\times \;10^{-3}$	5.53 $\times \;10^{-2}$	5	14
GSE29615_CTRL_VS_DAY3				
_LAIV_IFLU_VACCINE_PBMC_DN	1.26 $\times \;10^{-5}$	6.12 $\times \;10^{-2}$	0	194
BIOCARTA_NFAT_PATHWAY	5.41 $\times \;10^{-3}$	6.87 $\times \;10^{-2}$	18	51
GSE1112_OT1_CD8AB_VS_HY				
_CD8AA_THYMOCYTE_RTOC_CULTURE_DN	3.54 $\times \;10^{-5}$	8.00 $\times \;10^{-2}$	0	194
GSE12963_UNINF_VS_ENV_AND_NEF				
_DEFICIENT_HIV1_INF_CD4_TCELL_DN	4.93 $\times \;10^{-5}$	8.00 $\times \;10^{-2}$	0	146
BIOCARTA_GATA3_PATHWAY	9.52 $\times \;10^{-3}$	9.93 $\times \;10^{-2}$	16	14
KEGG_RIG_I_LIKE_RECEPTOR				
_SIGNALING_PATHWAY	0.22 $\times \;10^{-2}$	1.15 $\times \;10^{-1}$	3	71
BIOCARTA_LYM_PATHWAY	2.23 $\times \;10^{-3}$	1.58 $\times \;10^{-1}$	5	14
BIOCARTA_CDMAC_PATHWAY	2.40 $\times \;10^{-2}$	1.79 $\times \;10^{-1}$	12	16
GARGALOVIC_RESPONSE_TO				
_OXIDIZED_PHOSPHOLIPIDS_CYAN_UP	2.38 $\times \;10^{-4}$	1.87 $\times \;10^{-1}$	0	17
BIOCARTA_NKCELLS_PATHWAY	2.84 $\times \;10^{-2}$	1.90 $\times \;10^{-1}$	6	20
BIOCARTA_MSP_PATHWAY	3.20 $\times \;10^{-2}$	2.01 $\times \;10^{-1}$	2	6
BIOCARTA_GRANULOCYTES_PATHWAY	4.03 $\times \;10^{-2}$	2.26 $\times \;10^{-1}$	6	15
BIOCARTA_NEUTROPHIL_PATHWAY	4.02 $\times \;10^{-2}$	2.26 $\times \;10^{-1}$	4	8
BIOCARTA_IL17_PATHWAY	4.31 $\times \;10^{-2}$	2.29 $\times \;10^{-1}$	7	15
BIOCARTA_RNA_PATHWAY	4.28 $\times \;10^{-2}$	2.29 $\times \;10^{-1}$	5	10
PID_AVB3_INTEGRIN_PATHWAY	1.20 $\times \;10^{-2}$	2.42 $\times \;10^{-1}$	4	74
GOBP_NEGATIVE_REGULATION_OF				
_INTERLEUKIN_5_PRODUCTION	6.38 $\times \;10^{-4}$	2.42 $\times \;10^{-1}$	0	8

**Table 4:** The 22 significant gene sets that are related to developmental processes, sorted by their FDR (FDR cut-off < 0.25 and *p*-value < 0.05). Each significant developmental gene set is described by its *p*-value, FDR, degree, i.e., number of edges within the developmental ALS enrichment network containing gene sets with FDR < 0.25, and their corresponding number of genes. FDR: false discovery rate.

Gene Sets	*p*-Value	FDR	Degree	No. Genes
BIOCARTA_MPR_PATHWAY	6.27 $\times \;10^{-5}$	9.15 $\times \;10^{-3}$	18	21
BIOCARTA_SHH_PATHWAY	6.60 $\times \;10^{-4}$	4.03 $\times \;10^{-2}$	18	16
PID_NCADHERIN_PATHWAY	5.77 $\times \;10^{-4}$	5.66 $\times \;10^{-2}$	4	33
BIOCARTA_AGR_PATHWAY	7.82 $\times \;10^{-3}$	8.78 $\times \;10^{-2}$	3	33
PID_LYMPH_ANGIOGENESIS_PATHWAY	1.45 $\times \;10^{-3}$	9.46 $\times \;10^{-2}$	14	25
HP_ABNORMAL_RIB_CAGE_MORPHOLOGY	6.03 $\times \;10^{-5}$	1.02 $\times \;10^{-1}$	9	354
HP_THORACIC_HYPOPLASIA	5.01 $\times \;10^{-5}$	1.02 $\times \;10^{-1}$	3	138
HP_ONYCHOLYSIS	5.21 $\times \;10^{-5}$	1.02 $\times \;10^{-1}$	2	16
BIOCARTA_PTC1_PATHWAY	1.17 $\times \;10^{-2}$	1.09 $\times \;10^{-1}$	3	11
GOBP_MESODERM_DEVELOPMENT	7.52 $\times \;10^{-5}$	1.11 $\times \;10^{-1}$	6	132
GOBP_MESODERM_MORPHOGENESIS	1.65 $\times \;10^{-5}$	1.11 $\times \;10^{-1}$	5	75
GOBP_ANTEROGRADE_DENDRITIC_TRANSPORT				
_OF_NEUROTRANSMITTER_RECEPTOR_COMPLEX	5.60 $\times \;10^{-5}$	1.11 $\times \;10^{-1}$	1	5
KEGG_HEMATOPOIETIC_CELL_LINEAGE	3.68 $\times \;10^{-3}$	1.15 $\times \;10^{-1}$	4	87
HP_THORACIC_DYSPLASIA	9.63 $\times \;10^{-5}$	1.22 $\times \;10^{-1}$	3	6
NKX2_3_TARGET_GENES	2.65 $\times \;10^{-4}$	1.36 $\times \;10^{-1}$	0	544
GOBP_CARDIAC_MUSCLE_CELL_FATE				
_COMMITMENT	1.99 $\times \;10^{-4}$	1.90 $\times \;10^{-1}$	4	11
GOBP_GASTRULATION	5.13 $\times \;10^{-4}$	2.18 $\times \;10^{-1}$	10	190
GOBP_FORMATION_OF_PRIMARY_GERM_LAYER	4.68 $\times \;10^{-4}$	2.18 $\times \;10^{-1}$	10	123
HP_ABNORMALITY_OF_THE_RIBS	2.65 $\times \;10^{-4}$	2.24 $\times \;10^{-1}$	4	292
PID_AVB3_INTEGRIN_PATHWAY	1.20 $\times \;10^{-2}$	2.36 $\times \;10^{-1}$	7	74
GOBP_CHORIO_ALLANTOIC_FUSION	7.28 $\times \;10^{-4}$	2.42 $\times \;10^{-1}$	4	7
BIOCARTA_KERATINOCYTE_PATHWAY	4.95 $\times \;10^{-2}$	2.46 $\times \;10^{-1}$	10	46

**Table 5:** The significant first neighbors of the BioCarta mPR and Shh pathways, sorted by their FDR (FDR cut-off < 0.25 and *p*-value < 0.05). FDR: false discovery rate.

Overlapping Neighbors	*p*-Value	FDR
BIOCARTA_MPR_PATHWAY	6.27 $\times \;10^{-5}$	9.15 $\times \;10^{-3}$
BIOCARTA_CSK_PATHWAY	5.02 $\times \;10^{-5}$	9.15 $\times \;10^{-3}$
GOMF_CYCLIC_NUCLEOTIDE_DEPENDENT		
_PROTEIN_KINASE_ACTIVITY	6.70 $\times \;10^{-5}$	3.88 $\times \;10^{-2}$
GOMF_CYCLIC_NUCLEOTIDE_BINDING	6.59 $\times \;10^{-5}$	3.88 $\times \;10^{-2}$
BIOCARTA_CREB_PATHWAY	4.05 $\times \;10^{-4}$	3.94 $\times \;10^{-2}$
BIOCARTA_DREAM_PATHWAY	1.10 $\times \;10^{-3}$	4.03 $\times \;10^{-2}$
BIOCARTA_SHH_PATHWAY	6.60 $\times \;10^{-4}$	4.03 $\times \;10^{-2}$
BIOCARTA_BAD_PATHWAY	8.07 $\times \;10^{-4}$	4.03 $\times \;10^{-2}$
BIOCARTA_IGF1R_PATHWAY	9.71 $\times \;10^{-4}$	4.03 $\times \;10^{-2}$
BIOCARTA_CFTR_PATHWAY	1.81 $\times \;10^{-3}$	4.63 $\times \;10^{-2}$
BIOCARTA_CK1_PATHWAY	1.71 $\times \;10^{-3}$	4.63 $\times \;10^{-2}$
BIOCARTA_PPARA_PATHWAY	1.71 $\times \;10^{-3}$	4.63 $\times \;10^{-2}$
BIOCARTA_AGPCR_PATHWAY	2.56 $\times \;10^{-3}$	4.85 $\times \;10^{-2}$
BIOCARTA_STATHMIN_PATHWAY	2.30 $\times \;10^{-3}$	4.85 $\times \;10^{-2}$
BIOCARTA_VIP_PATHWAY	2.87 $\times \;10^{-3}$	4.93 $\times \;10^{-2}$
HP_ABNORMAL_RIB_CAGE_MORPHOLOGY	6.03 $\times \;10^{-5}$	1.02 $\times \;10^{-1}$
BIOCARTA_PTC1_PATHWAY	1.17 $\times \;10^{-2}$	1.09 $\times \;10^{-1}$

**Table 6:** The 19 significant gene sets that are related to nervous system processes, sorted by their FDR (FDR cut-off < 0.25 and *p*-value < 0.05). Each gene set is also described by its degree in the nervous system-specific subnetwork, i.e., the number of undirected edges of each gene set/node and the number of genes it contains. FDR: false discovery rate.

Gene Sets	*p*-Value	FDR	Degree	No. Genes
BIOCARTA_CREB_PATHWAY	4.05 $\times \;10^{-4}$	3.94 $\times \;10^{-2}$	21	22
BIOCARTA_SHH_PATHWAY	6.60 $\times \;10^{-4}$	4.03 $\times \;10^{-2}$	16	16
BIOCARTA_DREAM_PATHWAY	1.10 $\times \;10^{-3}$	4.03 $\times \;10^{-2}$	19	13
BIOCARTA_CK1_PATHWAY	1.70 $\times \;10^{-3}$	4.63 $\times \;10^{-2}$	16	16
BIOCARTA_AGPCR_PATHWAY	2.56 $\times \;10^{-3}$	4.85 $\times \;10^{-2}$	18	11
BIOCARTA_PRION_PATHWAY	3.36 $\times \;10^{-3}$	5.42 $\times \;10^{-2}$	1	12
BIOCARTA_NOS1_PATHWAY	3.95 $\times \;10^{-3}$	5.53 $\times \;10^{-2}$	18	21
PID_NCADHERIN_PATHWAY	5.77 $\times \;10^{-4}$	5.66 $\times \;10^{-2}$	7	33
BIOCARTA_NFAT_PATHWAY	5.41 $\times \;10^{-3}$	6.87 $\times \;10^{-2}$	20	51
BIOCARTA_AGR_PATHWAY	7.82 $\times \;10^{-3}$	8.78 $\times \;10^{-2}$	4	33
GOBP_ANTEROGRADE_DENDRITIC				
_TRANSPORT_OF_NEUROTRANSMITTER				
_RECEPTOR_COMPLEX	5.60 $\times \;10^{-5}$	1.11 $\times \;10^{-1}$	1	5
BIOCARTA_TRKA_PATHWAY	1.92 $\times \;10^{-2}$	1.57 $\times \;10^{-1}$	11	14
BIOCARTA_CB1R_PATHWAY	2.19 $\times \;10^{-2}$	1.69 $\times \;10^{-1}$	1	7
BIOCARTA_PDZS_PATHWAY	2.53 $\times \;10^{-2}$	1.80 $\times \;10^{-1}$	0	18
DIERICK_SEROTONIN_FUNCTION_GENES	2.77 $\times \;10^{-4}$	1.87 $\times \;10^{-1}$	0	7
GOBP_VESICLE_MEDIATED_TRANSPORT				
_TO_THE_PLASMA_MEMBRANE	3.83 $\times \;10^{-4}$	2.18 $\times \;10^{-1}$	0	140
BIOCARTA_ERK5_PATHWAY	3.65 $\times \;10^{-2}$	2.19 $\times \;10^{-1}$	12	14
BIOCARTA_MAL_PATHWAY	4.39 $\times \;10^{-2}$	2.29 $\times \;10^{-1}$	10	19
BIOCARTA_NGF_PATHWAY	5.07 $\times \;10^{-2}$	2.47 $\times \;10^{-1}$	12	20

**Table 7:** The 13 significant gene sets that are related to muscle processes, sorted by their FDR (FDR cut-off < 0.25 and *p*-value < 0.05). Each gene set is also described by its degree in the muscle-specific subnetwork, i.e., the number of undirected edges of each gene set/node and the number of genes it contains. FDR: false discovery rate.

Gene Sets	*p*-Value	FDR	Degree	No. Genes
BIOCARTA_IGF1R_PATHWAY	9.71 $\times \;10^{-4}$	4.03 $\times \;10^{-2}$	19	23
PID_NCADHERIN_PATHWAY	5.77 $\times \;10^{-4}$	5.66 $\times \;10^{-2}$	3	33
BIOCARTA_NFAT_PATHWAY	5.41 $\times \;10^{-3}$	6.87 $\times \;10^{-2}$	18	51
BIOCARTA_AT1R_PATHWAY	6.55 $\times \;10^{-3}$	7.65 $\times \;10^{-2}$	13	27
BIOCARTA_AGR_PATHWAY	7.82 $\times \;10^{-3}$	8.78 $\times \;10^{-2}$	4	33
BIOCARTA_IGF1MTOR_PATHWAY	1.21 $\times \;10^{-2}$	1.09 $\times \;10^{-1}$	4	19
KEGG_BIOSYNTHESIS_OF				
_UNSATURATED_FATTY_ACIDS	2.59 $\times \;10^{-3}$	1.15 $\times \;10^{-1}$	0	22
HP_TORSADE_DE_POINTES	1.57 $\times \;10^{-4}$	1.59 $\times \;10^{-1}$	0	24
GOBP_CARDIAC_MUSCLE_CELL				
_FATE_COMMITMENT	1.99 $\times \;10^{-4}$	1.90 $\times \;10^{-1}$	0	11
GOBP_VASCULAR_ASSOCIATED_SMOOTH				
_MUSCLE_CELL_MIGRATION	3.42 $\times \;10^{-4}$	2.18 $\times \;10^{-1}$	2	45
GOBP_MUSCLE_CELL_MIGRATION	4.75 $\times \;10^{-4}$	2.18 $\times \;10^{-1}$	3	104
BIOCARTA_MAL_PATHWAY	4.39 $\times \;10^{-2}$	2.29 $\times \;10^{-1}$	9	19
BIOCARTA_MYOSIN_PATHWAY	4.81 $\times \;10^{-2}$	2.46 $\times \;10^{-1}$	8	11

**Table 8:** The 9 significant gene sets that are related to lipid metabolism, sorted by their FDR (FDR cut-off < 0.25 and *p*-value < 0.05). Each gene set is also described by its degree in the lipid metabolism subnetwork, i.e., the number of undirected edges of each gene set/node and the number of genes it contains. FDR: false discovery rate.

Gene Sets	*p*-Value	FDR	Degree	No. Genes
BIOCARTA_CFTR_PATHWAY	1.81 $\times \;10^{-3}$	4.63 $\times \;10^{-2}$	14	11
BIOCARTA_PPARA_PATHWAY	1.71 $\times \;10^{-3}$	4.63 $\times \;10^{-2}$	14	52
GOBP_UNSATURATED_FATTY				
_ACID_BIOSYNTHETIC_PROCESS	4.90 $\times \;10^{-5}$	1.11 $\times \;10^{-1}$	1	52
KEGG_BIOSYNTHESIS_OF				
_UNSATURATED_FATTY_ACIDS	2.59 $\times \;10^{-3}$	1.15 $\times \;10^{-1}$	1	22
PID_S1P_META_PATHWAY	2.55 $\times \;10^{-3}$	1.25 $\times \;10^{-1}$	1	21
PID_S1P_S1P2_PATHWAY	3.92 $\times \;10^{-3}$	1.49 $\times \;10^{-1}$	5	24
GOBP_VESICLE_MEDIATED_TRANSPORT				
_TO_THE_PLASMA_MEMBRANE	3.83 $\times \;10^{-4}$	2.18 $\times \;10^{-1}$	0	140
GOBP_ADIPOSE_TISSUE_DEVELOPMENT	4.76 $\times \;10^{-4}$	2.18 $\times \;10^{-1}$	0	46
BURTON_ADIPOGENESIS_2	6.41 $\times \;10^{-4}$	2.41 $\times \;10^{-1}$	0	71

**Table 9:** The 4 significant interactions of pairs of ALS-associated gene sets, sorted by their *p*-value. Each interaction is described by the pair of ALS-associated gene sets, the number of overlapping genes between the two gene sets and their MAGMA interaction *p*-value.

Gene Set A	Gene Set B	Overlapping Genes	*p*-Value
BIOCARTA_PPARA_PATHWAY	BIOCARTA_GPCR_PATHWAY	10	1.36 $\times \;10^{-2}$
KEGG_HEMATOPOIETIC_CELL_LINEAGE	KEGG_ECM_RECEPTOR_INTERACTION	14	1.95 $\times \;10^{-2}$
BIOCARTA_PPARA_PATHWAY	BIOCARTA_CREB_PATHWAY	12	3.15 $\times \;10^{-2}$
KEGG_GAP_JUNCTION	BIOCARTA_GPCR_PATHWAY	10	4.67 $\times \;10^{-2}$

**Table 10:** Summary of the total 31,454 collected gene sets, described by their main and sub-categories used in this study, mined from Molecular Signatures Database (MSigDB v7.5). TFT: transcription factor targets.

Main Category	Sub-Categories	No. Gene Sets
C2: curated gene sets	CGP: chemical and genetic perturbations	3383
	Canonical Pathways: BioCarta	292
	Canonical Pathways: KEGG	186
	Canonical Pathways: PID	196
	Canonical Pathways: REACTOME	1615
	Canonical Pathways: WikiPathways	664
C3: regulatory target gene sets	miRDB subset of MIR	2377
	MIR_Legacy subset of MIR	221
	GTRD subset of TFT	518
	TFT_Legacy subset of TFT	610
C5: ontology gene sets	Gene Ontology: Biological Process	7658
	Gene Ontology: Cellular Component	1006
	Gene Ontology: Molecular Function	1738
	HPO: Human Phenotype Ontology	5071
C7: immunologic signature gene sets	ImmuneSigDB subset of C7	4872
	VAX: vaccine response gene sets	347
C8: cell type signature gene sets	-	700

## Data Availability

The data analyzed in the current publication are from dbGaP accessions numbers phs000101.v5.p1 and phs000428.v2.p2.

## Supplementary Materials

The following supporting information can be downloaded at: https://www.mdpi.com/article/10.3390/ijms24044021/s1. Figures S1–S6: High-resolution versions of Figure 2, Figure 3, Figure 4, Figure 5, Figure 6 and Figure 7, respectively. Table S1: Gene-level summary statistics for the genes of each of the gene sets (those gene sets having FDR < 0.05). Multiple-testing adjusted *p*-values (FDR) are given for each gene’s association to the ALS phenotype.

## References

[1] Niedermeyer S., Murn M., Choi P.J. (2019). Respiratory Failure in Amyotrophic Lateral Sclerosis. Chest.

[2] Chiò A., Logroscino G., Traynor B., Collins J., Simeone J., Goldstein L., White L. (2013). Global Epidemiology of Amyotrophic Lateral Sclerosis: A Systematic Review of the Published Literature. Neuroepidemiology.

[3] Al-Chalabi A., Van Den Berg L.H., Veldink J. (2017). Gene discovery in amyotrophic lateral sclerosis: Implications for clinical management. Nat. Rev. Neurol..

[4] Chiò A., Logroscino G., Hardiman O., Swingler R., Mitchell D., Beghi E., Traynor B.G. (2009). Prognostic factors in ALS: A critical review. Amyotroph Lateral Scler..

[5] Arthur K.C., Calvo A., Price T.R., Geiger J.T., Chiò A., Traynor B.J. (2016). Projected increase in amyotrophic lateral sclerosis from 2015 to 2040. Nat. Commun..

[6] Rowland L.P., Shneider N.A. (2001). Amyotrophic Lateral Sclerosis. N. Engl. J. Med..

[7] Vijayakumar U.G., Milla V., Stafford M.Y.C., Bjourson A.J., Duddy W., Duguez S.M.R. (2019). A systematic review of suggested molecular strata, biomarkers and their tissue sources in ALS. Front. Neurol..

[8] Turner M.R., Al-Chalabi A., Chio A., Hardiman O., Kiernan M.C., Rohrer J.D., Rowe J., Seeley W., Talbot K. (2017). Genetic screening in sporadic ALS and FTD. J. Neurol. Neurosurg. Psychiatry.

[9] Nicolas A., Kenna K., Renton A.E., Ticozzi N., Faghri F., Chia R., Dominov J.A., Kenna B.J., Nalls M.A., Keagle P. (2018). Genome-wide Analyses Identify KIF5A as a Novel ALS Gene. Neuron.

[10] Chia R., Chiò A., Traynor B.J. (2018). Novel genes associated with amyotrophic lateral sclerosis: Diagnostic and clinical implications. Lancet Neurol..

[11] Volk A.E., Weishaupt J.H., Andersen P.M., Ludolph A.C., Kubisch C. (2018). Current knowledge and recent insights into the genetic basis of amyotrophic lateral sclerosis. Med. Genet..

[12] Smukowski S.N., Maioli H., Latimer C.S., Bird T.D., Jayadev S., Valdmanis P.N. (2022). Progress in Amyotrophic Lateral Sclerosis Gene Discovery. Neurol. Genet..

[13] Zou Z.Y., Zhou Z.R., Che C.H., Liu C.Y., He R.L., Huang H.P. (2017). Genetic epidemiology of amyotrophic lateral sclerosis: A systematic review and meta-analysis. J. Neurol. Neurosurg. Psychiatry.

[14] Connolly O., Le Gall L., McCluskey G., Donaghy C.G., Duddy W.J., Duguez S. (2020). A Systematic Review of Genotype–Phenotype Correlation across Cohorts Having Causal Mutations of Different Genes in ALS. J. Pers. Med..

[15] McLaughlin L.R., Vajda A., Hardiman O. (2015). Heritability of amyotrophic lateral sclerosis insights from disparate numbers. JAMA Neurol..

[16] Vasilopoulou C., Morris A.P., Giannakopoulos G., Duguez S., Duddy W. (2020). What Can Machine Learning Approaches in Genomics Tell Us about the Molecular Basis of Amyotrophic Lateral Sclerosis?. J. Pers. Med..

[17] Gall L.L., Anakor E., Connolly O., Vijayakumar U.G., Duguez S. (2020). Molecular and cellular mechanisms affected in ALS. J. Pers. Med..

[18] Du Y., Wen Y., Guo X., Hao J., Wang W., He A., Fan Q., Li P., Liu L., Liang X. (2018). A Genome-wide Expression Association Analysis Identifies Genes and Pathways Associated with Amyotrophic Lateral Sclerosis. Cell. Mol. Neurobiol..

[19] De Leeuw C.A., Neale B.M., Heskes T., Posthuma D. (2016). The statistical properties of gene-set analysis. Nat. Rev. Genet..

[20] Vasilopoulou C., Duguez S., Duddy W. (2022). Genome-Wide Gene-Set Analysis Approaches in Amyotrophic Lateral Sclerosis. J. Pers. Med..

[21] Maleki F., Ovens K., Hogan D.J., Kusalik A.J. (2020). Gene Set Analysis: Challenges, Opportunities, and Future Research. Front. Genet..

[22] de Leeuw C.A., Mooij J.M., Heskes T., Posthuma D. (2015). MAGMA: Generalized Gene-Set Analysis of GWAS Data. PLoS Comput. Biol..

[23] de Leeuw C.A., Stringer S., Dekkers I.A., Heskes T., Posthuma D. (2018). Conditional and interaction gene-set analysis reveals novel functional pathways for blood pressure. Nat. Commun..

[24] Liberzon A., Birger C., Thorvaldsdóttir H., Ghandi M., Mesirov J.P., Tamayo P. (2015). The Molecular Signatures Database (MSigDB) hallmark gene set collection. Cell Syst..

[25] Subramanian A., Tamayo P., Mootha V.K., Mukherjee S., Ebert B.L., Gillette M.A., Paulovich A., Pomeroy S.L., Golub T.R., Lander E.S. (2005). Gene set enrichment analysis: A knowledge-based approach for interpreting genome-wide expression profiles. Proc. Natl. Acad. Sci. USA.

[26] Mailman M.D., Feolo M., Jin Y., Kimura M., Tryka K., Bagoutdinov R., Hao L., Kiang A., Paschall J., Phan L. (2007). The NCBI dbGaP database of genotypes and phenotypes. Nucleic Acids Res..

[27] Anderson C.A., Pettersson F.H., Clarke G.M., Cardon L.R., Morris P., Zondervan K.T. (2011). Data quality control in genetic case-control association studies. Nat. Protoc..

[28] Laurie C.C., Doheny K.F., Mirel D.B., Pugh E.W., Bierut J.L., Bhangale T., Boehm F., Caporaso N.E., Cornelis M.C., Edenberg H.J. (2011). Quality control and quality assurance in genotypic data for genome-wide association studies. Genet. Epidemiol..

[29] Li C.Y., Yang T.M., Ou R.W., Wei Q.Q., Shang H.F. (2021). Genome-wide genetic links between amyotrophic lateral sclerosis and autoimmune diseases. BMC Med..

[30] Renton A.E., Majounie E., Waite A., Simón-Sánchez J., Rollinson S., Gibbs J.R., Schymick J.C., Laaksovirta H., van Swieten J.C., Myllykangas L. (2011). A hexanucleotide repeat expansion in C9ORF72 is the cause of chromosome 9p21-linked ALS-FTD. Neuron.

[31] Van Es M.A., Veldink J.H., Saris C.G., Blauw H.M., Van Vught P.W., Birve A., Lemmens R., Schelhaas H.J., Groen E.J., Huisman M.H. (2009). Genome-wide association study identifies 19p13.3 (UNC13A) and 9p21.2 as susceptibility loci for sporadic amyotrophic lateral sclerosis. Nat. Genet..

[32] Vang T., Torgersen K.M., Sundvold V., Saxena M., Levy F.O., Skålhegg B.S., Hansson V., Mustelin T., Taskén K. (2001). Activation of the Cooh-Terminal Src Kinase (Csk) by Camp-Dependent Protein Kinase Inhibits Signaling through the T Cell Receptor. J. Exp. Med..

[33] Fife B.T., Bluestone J.A. (2008). Control of peripheral T-cell tolerance and autoimmunity via the CTLA-4 and PD-1 pathways. Immunol. Rev..

[34] Jansen M.I., Broome S.T., Castorina A. (2022). Exploring the Pro-Phagocytic and Anti-Inflammatory Functions of PACAP and VIP in Microglia: Implications for Multiple Sclerosis. Int. J. Mol. Sci..

[35] Macian F. (2005). NFAT proteins: Key regulators of T-cell development and function. Nat. Rev. Immunol..

[36] Murga-Zamalloa C., Wilcox R.A. (2020). GATA-3 in T-cell lymphoproliferative disorders. IUBMB Life.

[37] Hanssens L.S., Duchateau J., Casimir G.J. (2021). CFTR Protein: Not Just a Chloride Channel?. Cells.

[38] Yoshimura K., Nakamura H., Trapnell B.C., Chu C.S., Dakemans W., Pavirani A., Lecocq J.P., Crystal R.G. (1991). Expression of the cystic fibrosis transmembrane conductance regulator gene in cells of non-epithelial origin. Nucleic Acids Res..

[39] Ren Z., Yu Y., Chen C., Yang D., Ding T., Zhu L., Deng J., Xu Z. (2021). The Triangle Relationship Between Long Noncoding RNA, RIG-I-like Receptor Signaling Pathway, and Glycolysis. Front. Microbiol..

[40] Misra U.K., Gawdi G., Akabani G., Pizzo S.V. (2002). Cadmium-induced DNA synthesis and cell proliferation in macrophages: The role of intracellular calcium and signal transduction mechanisms. Cell. Signal..

[41] Cesaro T., Michiels T. (2021). Inhibition of PKR by Viruses. Front. Microbiol..

[42] Gargalovic P.S., Imura M., Zhang B., Gharavi N.M., Clark M.J., Pagnon J., Yang W.P., He A., Truong A., Patel S. (2006). Identification of inflammatory gene modules based on variations of human endothelial cell responses to oxidized lipids. Proc. Natl. Acad. Sci. USA.

[43] Freigang S. (2016). The regulation of inflammation by oxidized phospholipids. Eur. J. Immunol..

[44] McGeachy M.J., Cua D.J., Gaffen S.L. (2019). The IL-17 family of cytokines in health and disease. Immunity.

[45] Amatya N., Garg A.V., Gaffen S.L. (2017). IL-17 Signaling: The Yin and the Yang. Trends Immunol..

[46] Pelaia C., Paoletti G., Puggioni F., Racca F., Pelaia G., Canonica G.W., Heffler E. (2019). Interleukin-5 in the Pathophysiology of Severe Asthma. Front. Physiol..

[47] Lambrecht B.N., Hammad H. (2015). The immunology of asthma. Nat. Immunol..

[48] Mezu-Ndubuisi O.J., Maheshwari A. (2021). The role of integrins in inflammation and angiogenesis. Pediatr. Res..

[49] Holesh J.E., Bass A.N., Lord M. (2022). Physiology, Ovulation.

[50] Fair T., Lonergan P. (2012). The role of progesterone in oocyte acquisition of developmental competence. Reprod. Domest. Anim. Zuchthyg..

[51] Thomas P., Pang Y. (2012). Membrane Progesterone Receptors (mPRs): Evidence for Neuroprotective, Neurosteroid Signaling and Neuroendocrine Functions in Neuronal Cells. Neuroendocrinology.

[52] Choudhry Z., Rikani A.A., Choudhry A.M., Tariq S., Zakaria F., Asghar M.W., Sarfraz M.K., Haider K., Shafiq A.A., Mobassarah N.J. (2014). Sonic hedgehog signalling pathway: A complex network. Ann. Neurosci..

[53] Jha N.K., Chen W.C., Kumar S., Dubey R., Tsai L.W., Kar R., Jha S.K., Gupta P.K., Sharma A., Gundamaraju R. (2022). Molecular mechanisms of developmental pathways in neurological disorders: A pharmacological and therapeutic review. Open Biol..

[54] Echelard Y., Epstein D.J., St-Jacques B., Shen L., Mohler J., McMahon J.A., McMahon A.P. (1993). Sonic hedgehog, a member of a family of putative signaling molecules, is implicated in the regulation of CNS polarity. Cell.

[55] Maronde E. (2021). Cyclic Nucleotide (cNMP) Analogues: Past, Present and Future. Int. J. Mol. Sci..

[56] Linder J.U., Schultz J.E. (2010). Use of Chimeric Adenylyl Cyclases to Study Cyclic Nucleotide Signaling. Handbook of Cell Signaling.

[57] Atwood B.K., Lopez J., Wager-Miller J., Mackie K., Straiker A. (2011). Expression of G protein-coupled receptors and related proteins in HEK293, AtT20, BV2, and N18 cell lines as revealed by microarray analysis. BMC Genom..

[58] Cheng H.Y.M., Pitcher G.M., Laviolette S.R., Whishaw I.Q., Tong K.I., Kockeritz L.K., Wada T., Joza N.A., Crackower M., Goncalves J. (2002). DREAM is a critical transcriptional repressor for pain modulation. Cell.

[59] Steven A., Friedrich M., Jank P., Heimer N., Budczies J., Denkert C., Seliger B. (2020). What turns CREB on? And off? And why does it matter?. Cell. Mol. Life Sci..

[60] Jiang J. (2017). CK1 in Developmental signaling: Hedgehog and Wnt. Curr. Top. Dev. Biol..

[61] Luo R., Su L.Y., Li G., Yang J., Liu Q., Yang L.X., Zhang D.F., Zhou H., Xu M., Fan Y. (2020). Activation of PPARA-mediated autophagy reduces Alzheimer disease-like pathology and cognitive decline in a murine model. Autophagy.

[62] Brusés J.L. (2006). N-cadherin signaling in synapse formation and neuronal physiology. Mol. Neurobiol..

[63] Lelièvre E.C., Plestant C., Boscher C., Wolff E., Mège R.M., Birbes H. (2012). N-cadherin mediates neuronal cell survival through Bim down-regulation. PLoS ONE.

[64] Redies C., Treubert-Zimmermann U., Luo J. (2003). Cadherins as regulators for the emergence of neural nets from embryonic divisions. J. Physiol. Paris.

[65] Takeichi M., Matsunami H., Inoue T., Kimura Y., Suzuki S., Tanaka T. (1997). Roles of cadherins in patterning of the developing brain. Dev. Neurosci..

[66] Sun Z., Parrish A.R., Hill M.A., Meininger G.A. (2014). N-cadherin, a vascular smooth muscle cell-cell adhesion molecule: Function and signaling for vasomotor control. Microcirculation.

[67] Guntur A.R., Rosen C.J., Naski M.C. (2012). N-cadherin adherens junctions mediate osteogenesis through PI3K signaling. Bone.

[68] Kim H.J., Choi H.S., Park J.H., Kim M.J., Lee H.G., Petersen R.B., Kim Y.S., Park J.B., Choi E.K. (2017). Regulation of RhoA activity by the cellular prion protein. Cell Death Dis..

[69] Marrs G.S., Theisen C.S., Brusés J.L. (2009). N-cadherin modulates voltage activated calcium influx via RhoA, p120-catenin, and myosin-actin interaction. Mol. Cell. Neurosci..

[70] Muhr J., Ackerman K.M. (2022). Embryology, Gastrulation.

[71] Zhang Y., Ulvmar M.H., Stanczuk L., Martinez-Corral I., Frye M., Alitalo K., Mäkinen T. (2018). Heterogeneity in VEGFR3 levels drives lymphatic vessel hyperplasia through cell-autonomous and non-cell-autonomous mechanisms. Nat. Commun..

[72] Perez D.I., Gil C., Martinez A. (2011). Protein kinases CK1 and CK2 as new targets for neurodegenerative diseases. Med. Res. Rev..

[73] Kopp-Scheinpflug C., Forsythe I.D. (2021). Nitric Oxide Signaling in the Auditory Pathway. Front. Neural Circuits.

[74] Nagamoto-Combs K., Combs C.K. (2010). Microglial phenotype is regulated by activity of the transcription factor, NFAT (nuclear factor of activated T cells). J. Neurosci..

[75] Yoshida S., Hasegawa T. (2022). Deciphering the prion-like behavior of pathogenic protein aggregates in neurodegenerative diseases. Neurochem. Int..

[76] Polymenidou M., Cleveland D.W. (2011). The Seeds of Neurodegeneration: Prion-like Spreading in ALS. Cell.

[77] Gau D., Roy P. (2018). SRF’ing and SAP’ing—The role of MRTF proteins in cell migration. J. Cell Sci..

[78] Cen B., Selvaraj A., Prywes R. (2004). Myocardin/MKL family of SRF coactivators: Key regulators of immediate early and muscle specific gene expression. J. Cell. Biochem..

[79] Signaling I., Hakuno F., Takahashi S.I., Hakuno F., Takahashi S.I. (2018). 40 YEARS OF IGF1: IGF1 receptor signaling pathways. J. Mol. Endocrinol..

[80] Krauss R.S., Joseph G.A., Goel A.J. (2017). Keep Your Friends Close: Cell–Cell Contact and Skeletal Myogenesis. Cold Spring Harb. Perspect. Biol..

[81] Lehka L., Rȩdowicz M.J. (2020). Mechanisms regulating myoblast fusion: A multilevel interplay. Semin. Cell Dev. Biol..

[82] De Mello W.C., Danser A.H. (2000). Angiotensin II and the Heart. Hypertension.

[83] Islinger M., Voelkl A., Fahimi H.D., Schrader M. (2018). The peroxisome: An update on mysteries 2.0. Histochem. Cell Biol..

[84] Trachootham D., Lu W., Ogasawara M.A., Valle N.R.D., Huang P. (2008). Redox Regulation of Cell Survival. Antioxidants Redox Signal..

[85] Rakhshandehroo M., Knoch B., Müller M., Kersten S. (2010). Peroxisome proliferator-activated receptor alpha target genes. PPAR Res..

[86] Yoon M. (2009). The role of PPARalpha in lipid metabolism and obesity: Focusing on the effects of estrogen on PPARalpha actions. Pharmacol. Res..

[87] Zhang J.Q., Long X.Y., Xie Y., Zhao Z.H., Fang L.Z., Liu L., Fu W.P., Shu J.K., Wu J.H., Dai L.M. (2017). Relationship between PPAR*α* mRNA expression and mitochondrial respiratory function and ultrastructure of the skeletal muscle of patients with COPD. Bioengineered.

[88] Chen E.Y., Tan C.M., Kou Y., Duan Q., Wang Z., Meirelles G.V., Clark N.R., Ma’ayan A. (2013). Enrichr: Interactive and collaborative HTML5 gene list enrichment analysis tool. BMC Bioinform..

[89] Kuleshov M.V., Jones M.R., Rouillard A.D., Fernandez N.F., Duan Q., Wang Z., Koplev S., Jenkins S.L., Jagodnik K.M., Lachmann A. (2016). Enrichr: A comprehensive gene set enrichment analysis web server 2016 update. Nucleic Acids Res..

[90] Xie Z., Bailey A., Kuleshov M.V., Clarke D.J., Evangelista J.E., Jenkins S.L., Lachmann A., Wojciechowicz M.L., Kropiwnicki E., Jagodnik K.M. (2021). Gene Set Knowledge Discovery with Enrichr. Curr. Protoc..

[91] Charitou T., Bryan K., Lynn D.J. (2016). Using biological networks to integrate, visualize and analyze genomics data. Genet. Sel. Evol..

[92] Sun Y., Curle A.J., Haider A.M., Balmus G. (2020). The role of DNA damage response in amyotrophic lateral sclerosis. Essays Biochem..

[93] Herzog J.J., Xu W., Deshpande M., Rahman R., Suib H., Rodal A.A., Rosbash M., Paradis S. (2020). TDP-43 dysfunction restricts dendritic complexity by inhibiting CREB activation and altering gene expression. Proc. Natl. Acad. Sci. USA.

[94] Herzog J.J., Deshpande M., Shapiro L., Rodal A.A., Paradis S. (2017). TDP-43 misexpression causes defects in dendritic growth. Sci. Rep..

[95] Kweon J.H., Kim S., Lee S.B. (2017). The cellular basis of dendrite pathology in neurodegenerative diseases. BMB Rep..

[96] Stuart G.J., Spruston N. (2015). Dendritic integration: 60 years of progress. Nat. Neurosci..

[97] Catanese A., Rajkumar S., Sommer D., Freisem D., Wirth A., Aly A., Massa-L Opez D., Olivieri A., Torelli F., Ioannidis V. (2021). Synaptic disruption and CREB-regulated transcription are restored by K+ channel blockers in ALS. EMBO Mol. Med..

[98] Larrodé P., Calvo A.C., Moreno-Martínez L., De La Torre M., Moreno-García L., Molina N., Castiella T., Iñiguez C., Pascual L.F., Mena F.J.M. (2018). DREAM-dependent activation of astrocytes in amyotrophic lateral sclerosis. Mol. Neurobiol..

[99] Kametani F., Nonaka T., Suzuki T., Arai T., Dohmae N., Akiyama H., Hasegawa M. (2009). Identification of casein kinase-1 phosphorylation sites on TDP-43. Biochem. Biophys. Res. Commun..

[100] Xie Y., Luo X., He H., Tang M. (2021). Novel Insight Into the Role of Immune Dysregulation in Amyotrophic Lateral Sclerosis Based on Bioinformatic Analysis. Front. Neurosci..

[101] Morello G., Spampinato A.G., Cavallaro S. (2017). Neuroinflammation and ALS: Transcriptomic Insights into Molecular Disease Mechanisms and Therapeutic Targets. Mediat. Inflamm..

[102] Prinz M., Priller J. (2017). The role of peripheral immune cells in the CNS in steady state and disease. Nat. Neurosci..

[103] McCauley M.E., Baloh R.H. (2019). Inflammation in ALS/FTD pathogenesis. Acta Neuropathol..

[104] Geloso M.C., Corvino V., Marchese E., Serrano A., Michetti F., D’Ambrosi N. (2017). The dual role of microglia in ALS: Mechanisms and therapeutic approaches. Front. Aging Neurosci..

[105] Dong Y., Yong V.W. (2022). Oxidized phospholipids as novel mediators of neurodegeneration. Trends Neurosci..

[106] Catalá A. (2009). Lipid peroxidation of membrane phospholipids generates hydroxy-alkenals and oxidized phospholipids active in physiological and/or pathological conditions. Chem. Phys. Lipids.

[107] Lee D., Tomita Y., Allen W., Tsubota K., Negishi K., Kurihara T. (2021). PPAR*α* Modulation-Based Therapy in Central Nervous System Diseases. Life.

[108] Esmaeili M.A., Yadav S., Gupta R.K., Waggoner G.R., Deloach A., Calingasan N.Y., Flint Beal M., Kiaei M. (2016). Preferential PPAR-*α* activation reduces neuroinflammation, and blocks neurodegeneration in vivo. Hum. Mol. Genet..

[109] Spitaler M., Cantrell D.A. (2004). Protein kinase C and beyond. Nat. Immunol..

[110] Zhang H.L., Hu B.X., Li Z.L., Du T., Shan J.L., Ye Z.P., Peng X.D., Li X., Huang Y., Zhu X.Y. (2022). PKC*β*II phosphorylates ACSL4 to amplify lipid peroxidation to induce ferroptosis. Nat. Cell Biol..

[111] Zhou Z., Chen F., Zhong S., Zhou Y., Zhang R., Kang K., Zhang X., Xu Y., Zhao M., Zhao C. (2020). Molecular identification of protein kinase C beta in Alzheimer’s disease. Aging.

[112] Lo C., Cooper-Knock J., Garrard K., Martindale J., Williams T., Shaw P. (2013). Concurrent amyotrophic lateral sclerosis and cystic fibrosis supports common pathways of pathogenesis. Amyotroph. Lateral Scler. Front. Degener..

[113] De Nicola A.F., Meyer M., Garay L., Kruse M.S., Schumacher M., Guennoun R., Gonzalez Deniselle M.C. (2021). Progesterone and Allopregnanolone Neuroprotective Effects in the Wobbler Mouse Model of Amyotrophic Lateral Sclerosis. Cell. Mol. Neurobiol..

[114] Drannik A., Martin J., Peterson R., Ma X., Jiang F., Turnbull J. (2017). Cerebrospinal fluid from patients with amyotrophic lateral sclerosis inhibits sonic hedgehog function. PLoS ONE.

[115] Peterson R., Turnbull J. (2012). Sonic hedgehog is cytoprotective against oxidative challenge in a cellular model of amyotrophic lateral sclerosis. J. Mol. Neurosci..

[116] Liu Y.J., Ju T.C., Chen H.M., Jang Y.S., Lee L.M., Lai H.L., Tai H.C., Fang J.M., Lin Y.L., Tu P.H. (2015). Activation of AMP-activated protein kinase *α*1 mediates mislocalization of TDP-43 in amyotrophic lateral sclerosis. Hum. Mol. Genet..

[117] Hu J.H., Zhang H., Wagey R., Krieger C., Pelech S.L. (2003). Protein kinase and protein phosphatase expression in amyotrophic lateral sclerosis spinal cord. J. Neurochem..

[118] Vasilopoulou C., Duddy W., Wingfield B., Morris A.P. (2021). snpQT: Flexible, reproducible, and comprehensive quality control and imputation of genomic data. F1000Research.

[119] Purcell S., Neale B., Todd-Brown K., Thomas L., Ferreira M.A., Bender D., Maller J., Sklar P., De Bakker P.I., Daly M.J. (2007). PLINK: A tool set for whole-genome association and population-based linkage analyses. Am. J. Hum. Genet..

[120] Zuvich R.L., Armstrong L.L., Bielinski S.J., Bradford Y., Carlson C.S., Crawford D.C., Crenshaw A.T., de Andrade M., Doheny K.F., Haines J.L. (2011). Pitfalls of Merging GWAS Data: Lessons Learned in the eMERGE Network and Quality Control Procedures to Maintain High Data Quality. Genet. Epidemiol..

[121] McCarthy S., Das S., Kretzschmar W., Delaneau O., Wood A.R., Teumer A., Kang H.M., Fuchsberger C., Danecek P., Sharp K. (2016). A reference panel of 64,976 haplotypes for genotype imputation. Nat. Genet..

[122] Loh P.R., Danecek P., Palamara P.F., Fuchsberger C., Reshef Y.A., Finucane H.K., Schoenherr S., Forer L., McCarthy S., Abecasis G.R. (2016). Reference-based phasing using the Haplotype Reference Consortium panel. Nat. Genet..

[123] Durbin R. (2014). Efficient haplotype matching and storage using the positional Burrows-Wheeler transform (PBWT). Bioinformatics.

[124] Tsunoda T., Lathrop G.M., Sekine A., Yamada R., Takahashi A., Ohnishi Y., Tanaka T., Nakamura Y. (2004). Variation of gene-based SNPs and linkage disequilibrium patterns in the human genome. Hum. Mol. Genet..

[125] Shannon P., Markiel A., Ozier O., Baliga N.S., Wang J.T., Ramage D., Amin N., Schwikowski B., Ideker T. (2003). Cytoscape: A Software Environment for Integrated Models of Biomolecular Interaction Networks. Genome Res..

[126] Bateman A., Martin M.J., Orchard S., Magrane M., Agivetova R., Ahmad S., Alpi E., Bowler-Barnett E.H., Britto R., Bursteinas B. (2021). UniProt: The universal protein knowledgebase in 2021. Nucleic Acids Res..

[127] Safran M., Rosen N., Twik M., BarShir R., Stein T.I., Dahary D., Fishilevich S., Lancet D. (2021). The GeneCards Suite. Practical Guide to Life Science Databases.

[128] Carbon S., Ireland A., Mungall C.J., Shu S., Marshall B., Lewis S., Lomax J., Mungall C., Hitz B., Balakrishnan R. (2009). AmiGO: Online access to ontology and annotation data. Bioinformatics.

[129] Carbon S., Douglass E., Good B.M., Unni D.R., Harris N.L., Mungall C.J., Basu S., Chisholm R.L., Dodson R.J., Hartline E. (2021). The Gene Ontology resource: Enriching a GOld mine. Nucleic Acids Res..

[130] Ashburner M., Ball C.A., Blake J.A., Botstein D., Butler H., Cherry J.M., Davis A.P., Dolinski K., Dwight S.S., Eppig J.T. (2000). Gene ontology: Tool for the unification of biology. Nat. Genet..

[131] Kanehisa M., Furumichi M., Tanabe M., Sato Y., Morishima K. (2017). KEGG: New perspectives on genomes, pathways, diseases and drugs. Nucleic Acids Res..

[132] Jacobs B.M., Taylor T., Awad A., Baker D., Giovanonni G., Noyce A.J., Dobson R. (2020). Summary-data-based Mendelian randomization prioritizes potential druggable targets for multiple sclerosis. Brain Commun..

