# Supplementary figures and images for: Genome-Wide Gene-Set Analysis Identifies Molecular Mechanisms Associated with ALS
Source: Int J Mol Sci. 2023 Feb 16;24(4):4021. doi: 10.3390/ijms24044021 (PMC9966913; doi:10.3390/ijms24044021)

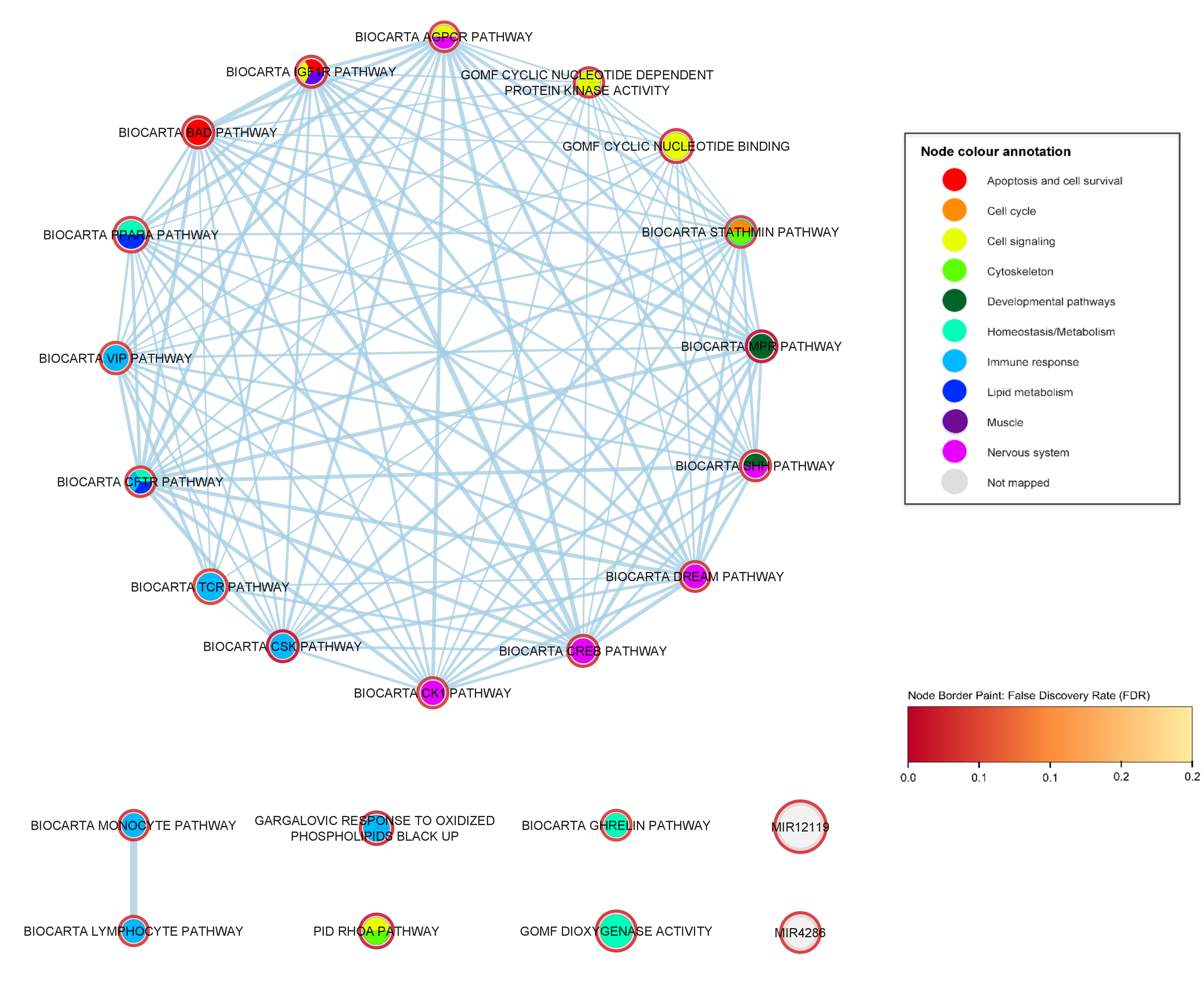

Supplement: Supplementary file 1 [file ijms-24-04021-s001.zip › Supplementary/Figure S1.png]

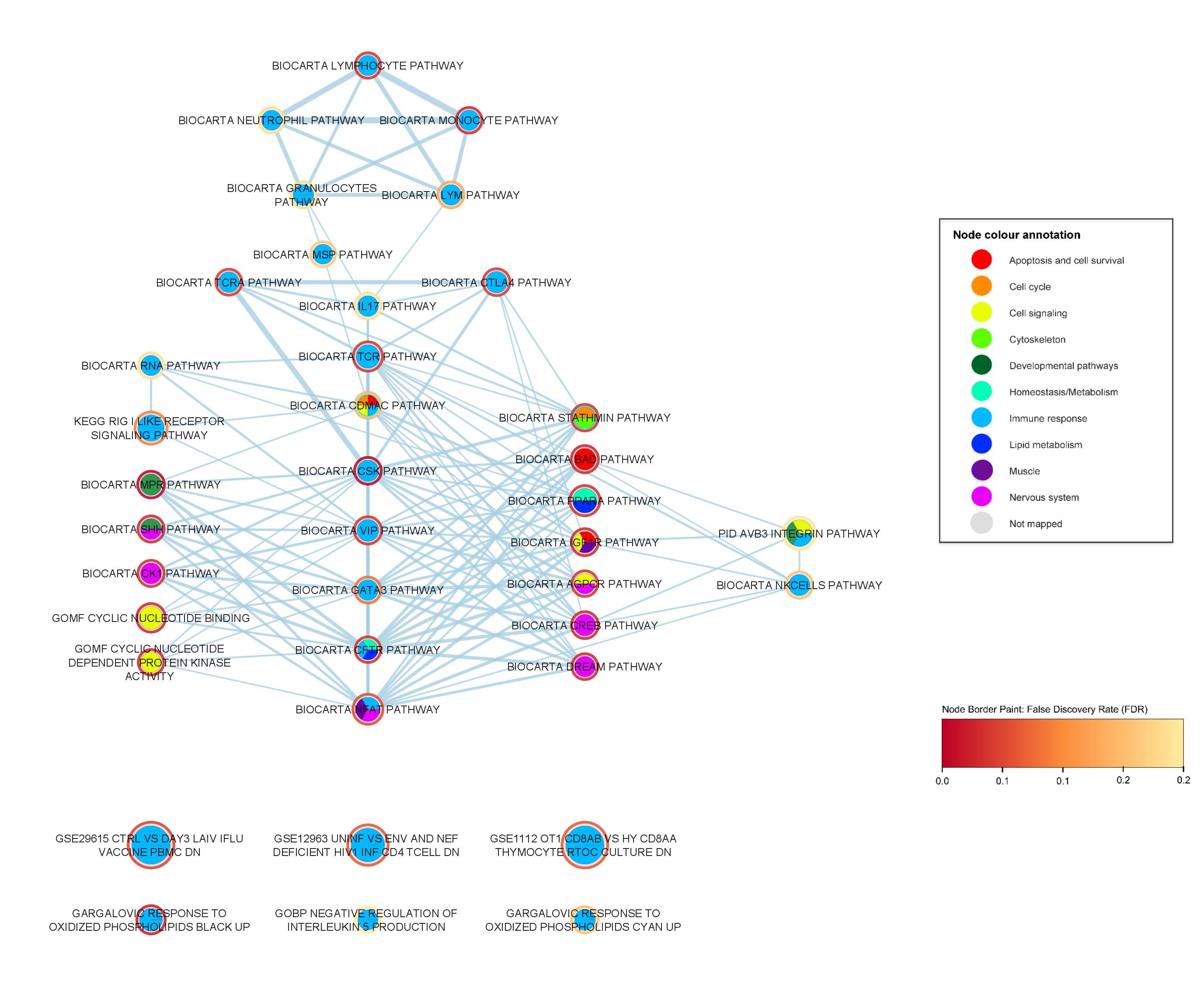

Supplement: Supplementary file 1 [file ijms-24-04021-s001.zip › Supplementary/Figure S2.png]

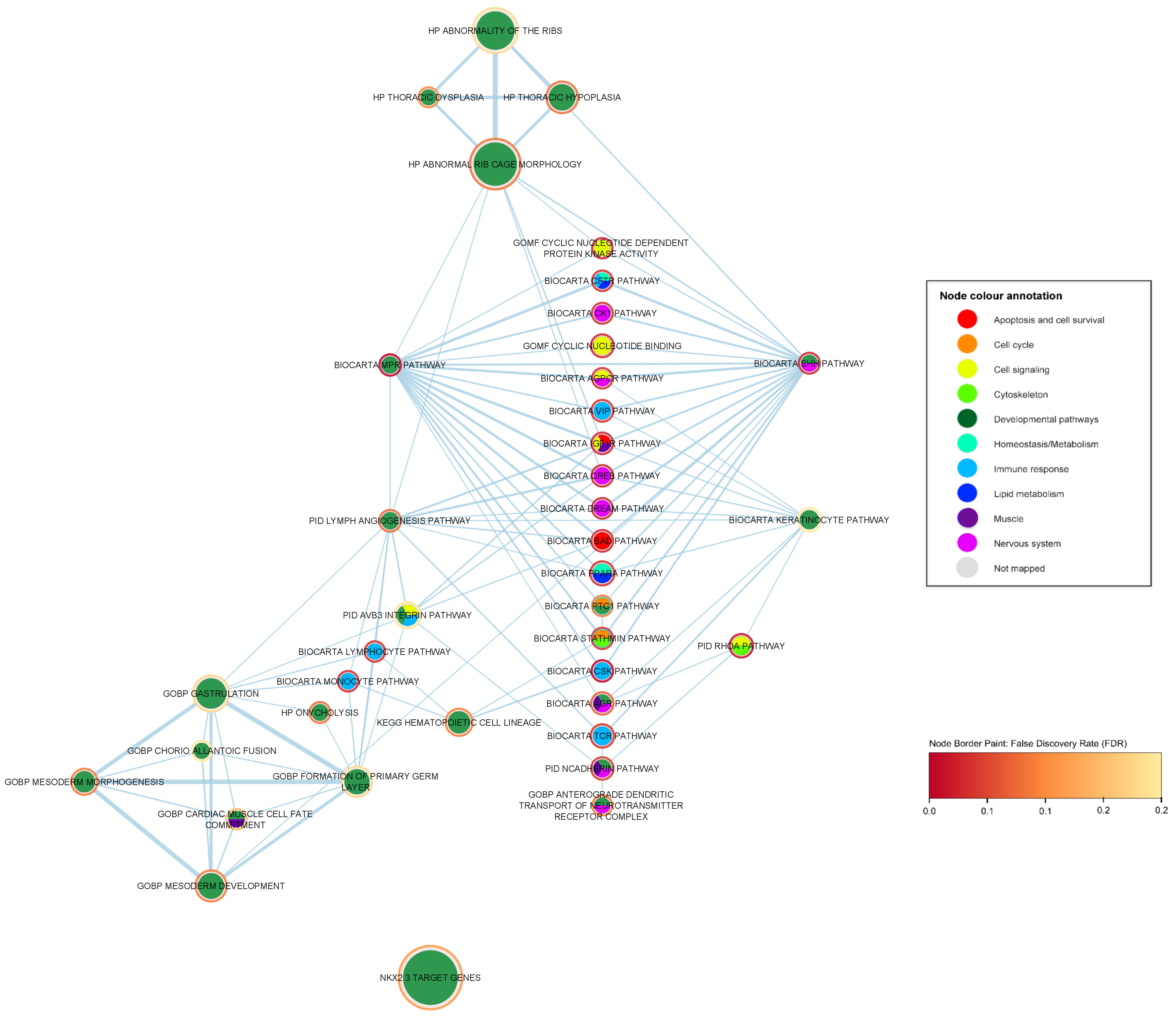

Supplement: Supplementary file 1 [file ijms-24-04021-s001.zip › Supplementary/Figure S3.png]

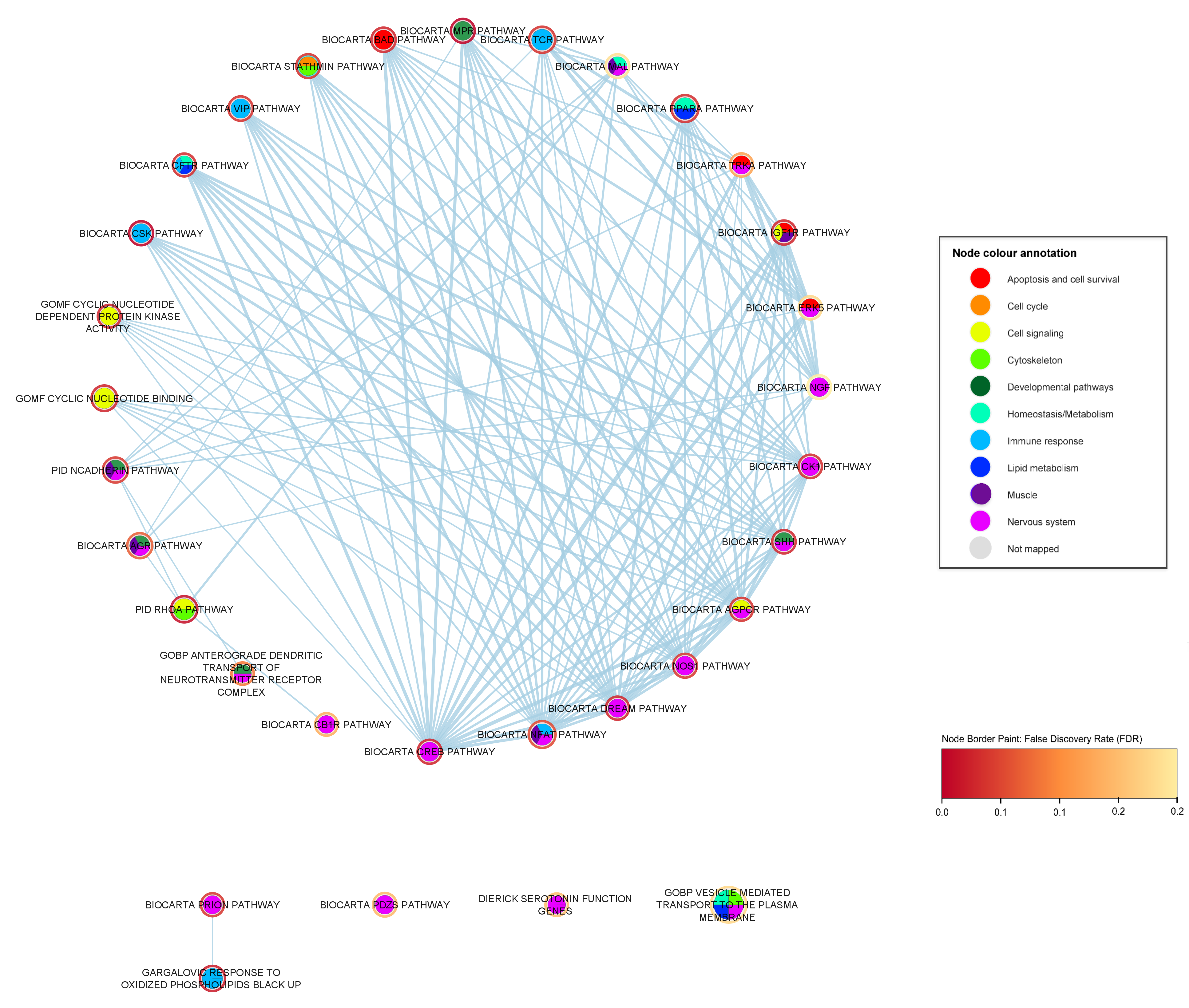

Supplement: Supplementary file 1 [file ijms-24-04021-s001.zip › Supplementary/Figure S4.png]

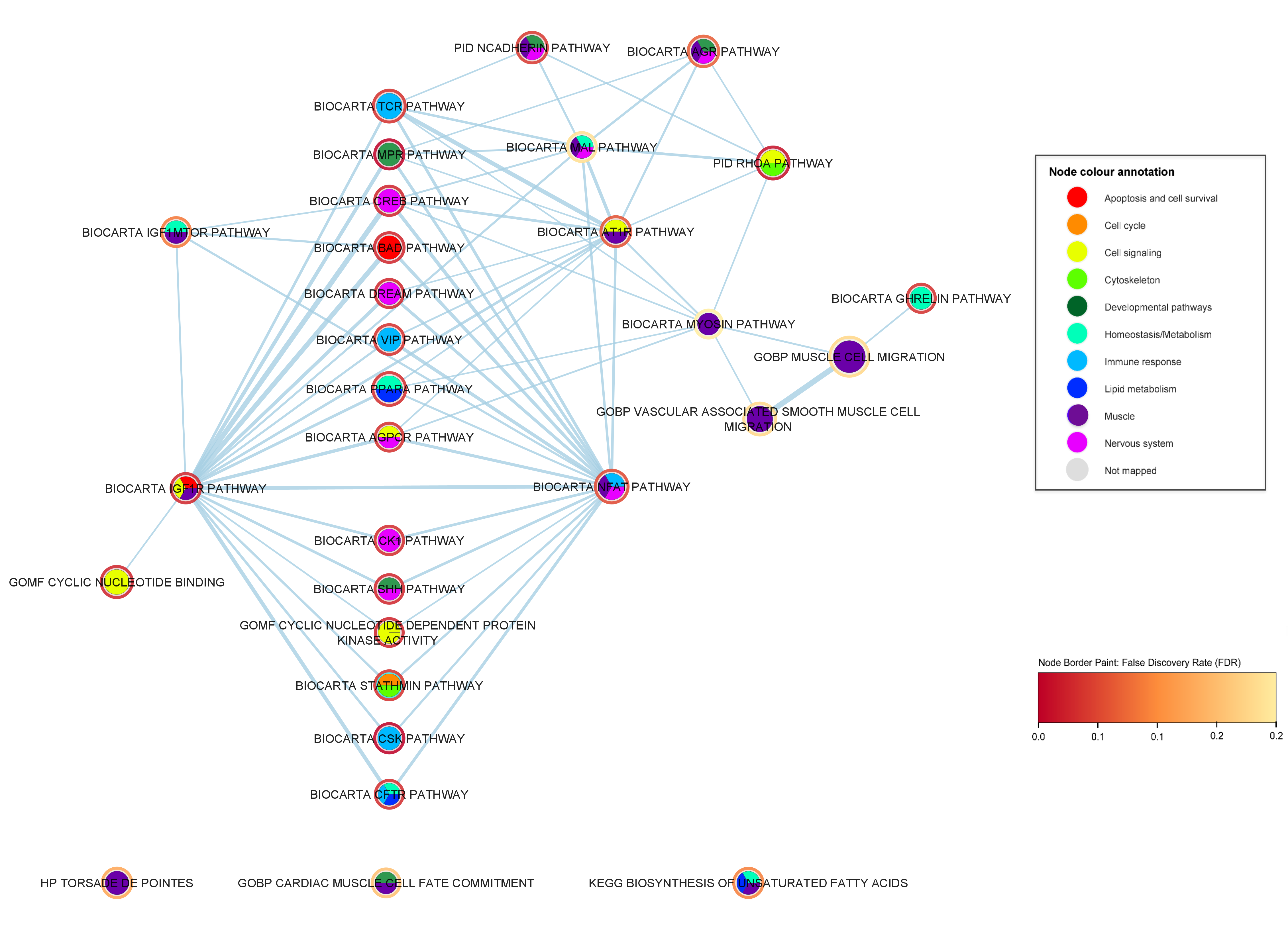

Supplement: Supplementary file 1 [file ijms-24-04021-s001.zip › Supplementary/Figure S5.png]

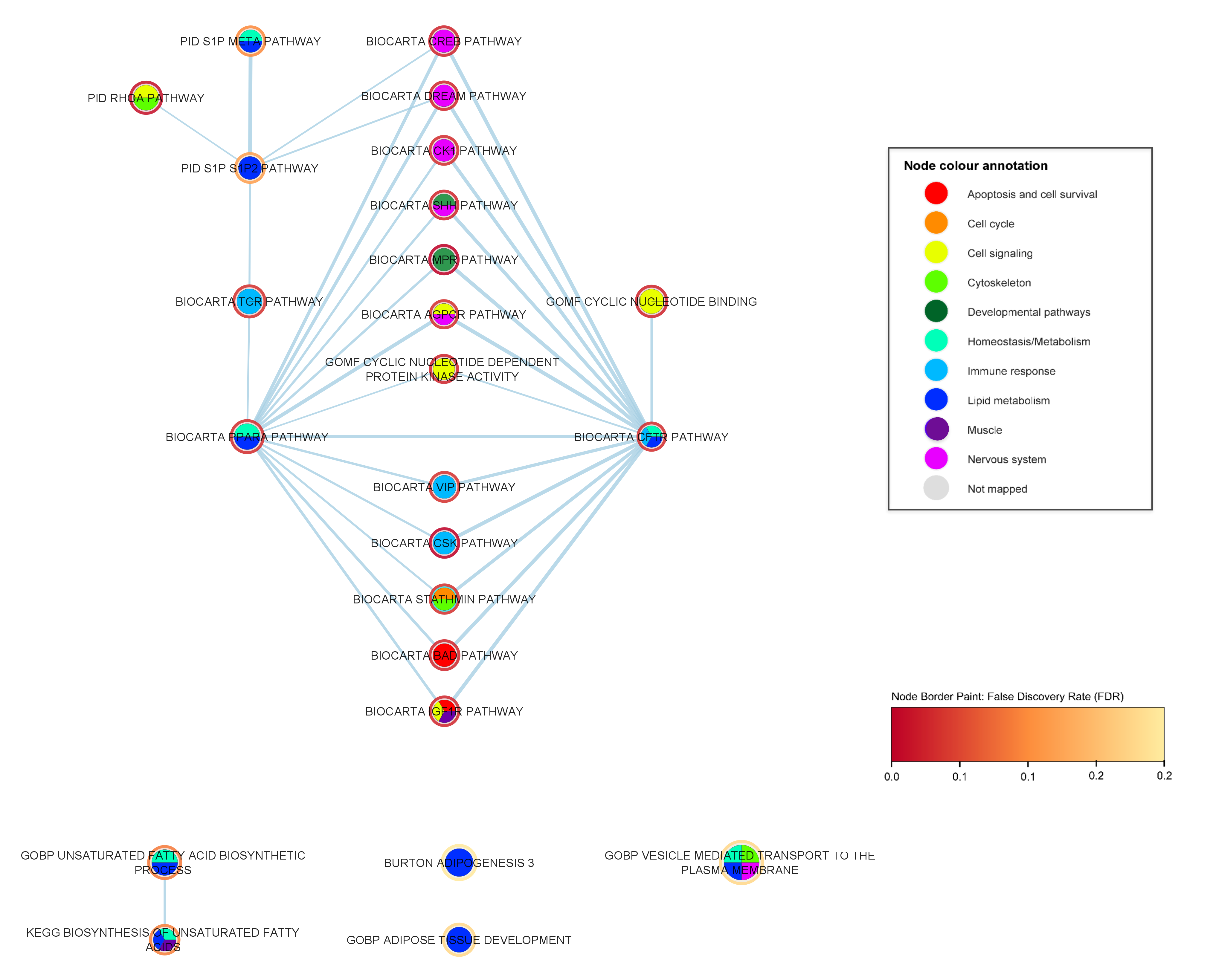

Supplement: Supplementary file 1 [file ijms-24-04021-s001.zip › Supplementary/Figure S6.png]
